# Characterization of FIREFLY, an Imaging Spectrometer Designed for Remote Sensing of Solar Induced Fluorescence

**DOI:** 10.3390/s20174682

**Published:** 2020-08-19

**Authors:** Ian Paynter, Bruce Cook, Lawrence Corp, Jyoteshwar Nagol, Joel McCorkel

**Affiliations:** 1 Universities Space Research Association, Columbia, MD 21046, USA; ian.paynter@nasa.gov; 2 Earth Sciences, NASA Goddard Space Flight Center, Greenbelt, MD 20771, USA; jyothy.nagol@gmail.com (J.N.); joel.mccorkel@nasa.gov (J.M.); 3 Science Systems & Applications Inc., Lanham, MD 20771, USA; lawrence.a.corp@nasa.gov; 4 Department of Geographical Sciences, University of Maryland, College Park, MD 20771, USA

**Keywords:** fluorescence, spectroscopy, remote sensing

## Abstract

Solar induced fluorescence (SIF) is an ecological variable of interest to remote sensing retrievals, as it is directly related to vegetation composition and condition. FIREFLY (fluorescence imaging of red and far-red light yield) is a high performance spectrometer for estimating SIF. FIREFLY was flown in conjunction with NASA Goddard’s lidar, hyperspectral, and thermal (G-LiHT) instrument package in 2017, as a technology demonstration for airborne retrievals of SIF. Attributes of FIREFLY relevant to SIF retrieval, including detector response and linearity; full-width at half maximum (FWHM); stray light; dark current; and shot noise were characterized with a combination of observations from Goddard’s laser for absolute measurement of radiance calibration facility; an integrating sphere; controlled acquisitions of known targets; in-flight acquisitions; and forward modelling. FWHM, stray light, and dark current were found to be of acceptable magnitude, and characterized to within acceptable limits for SIF retrieval. FIREFLY observations were found to represent oxygen absorption features, along with a large number of solar absorption features. Shot noise was acceptable for direct SIF retrievals at native resolution, but indirect SIF retrievals from absorption features would require spatial aggregation, or repeated observations of targets.

## 1. Introduction

The FIREFLY (fluorescence imaging of red and far-red light yield) instrument is a high performance spectrometer for estimating solar induced fluorescence (SIF). FIREFLY was operated in tandem with NASA Goddard’s lidar, hyperspectral and thermal (G-LiHT) airborne imager [1] for flight operations in 2017 and 2018. SIF is the re-emittance, at longer wavelengths, of solar energy absorbed by plants. SIF occurs, alongside the production of heat, when absorption of light exceeds the utilization of light by photosynthesis [2]. As such, SIF is related to photosynthesis, and therefore gross primary productivity (GPP), mediated by the light use efficiency of the plant [3,4]. Therefore, measuring SIF can provide quantitative insight into GPP [2,5], species composition [3], phenology [6], and the physiological status of plants, including stress [3,6,7], as well as being useful for crop monitoring [8]. The history, theory, methodologies, applications, and current challenges facing the retrieval of SIF via remote sensing were the subject of a recent comprehensive review [9].

Since the ecological attributes and processes that can be inferred from SIF measurements are of interest at the global scale, and across spatial and temporal gradients, efforts have been made to retrieve SIF from remote sensing resources, particularly from spectroscopy [2,3,10]. Fluorescence retrievals have been reported from satellite-based instruments such as SCIAMACHY [11]; GOME-2 [12]; TANSO-FTS [10]; OCO-2 [7,13]; and TROPOMI [14], resulting in global fluorescence products [5,11,12,15]. SIF retrievals have also already been reported from airborne instruments including HyPlant [16] and the compact fluorescence imaging spectrometer (CFIS) [17].

**Figure 1:**
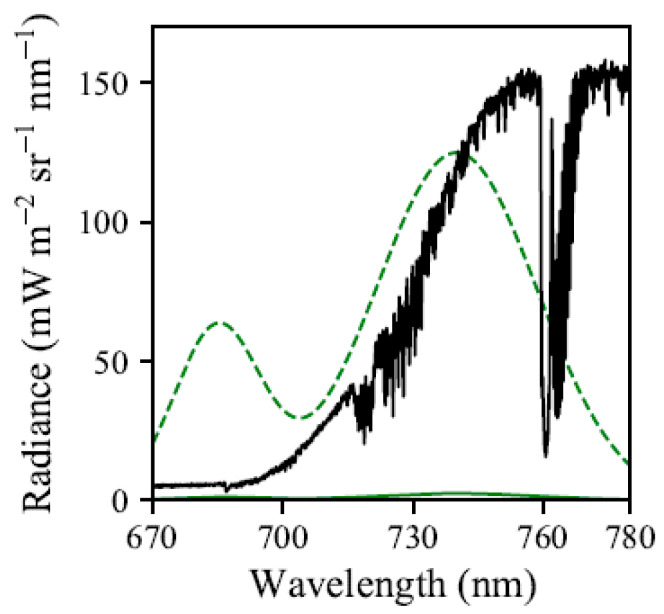
A sample of upwelling radiance of vegetation observed from fluorescence imaging of red and far-red light yield (FIREFLY) (black line), accompanied by an example SIF function to scale (green line), and the same solar induced fluorescence (SIF) function multiplied by a factor of 100 (green dashed line) to show the position of the peaks.

From the perspective of spectroscopy, fluorescence is both a relatively weak signal [3,18,19] and can comprise only a small portion of the energy retrieved by spectroscopy in the relevant wavelengths (Figure 1). SIF has been reported as approximately 1% of total absorbed solar energy [6,7,20], 2% in some regions [4,21], and 1–5% in the near infrared region [9,22]. The recent review of Mohammed et al. [9], depicted SIF with a peak of approximately 2.5 mW m ^−2^ sr ^−1^ nm ^−1^, and this representation is used as a benchmark to provide context in this study, and is shown in Figure 1.

**Figure 2:**
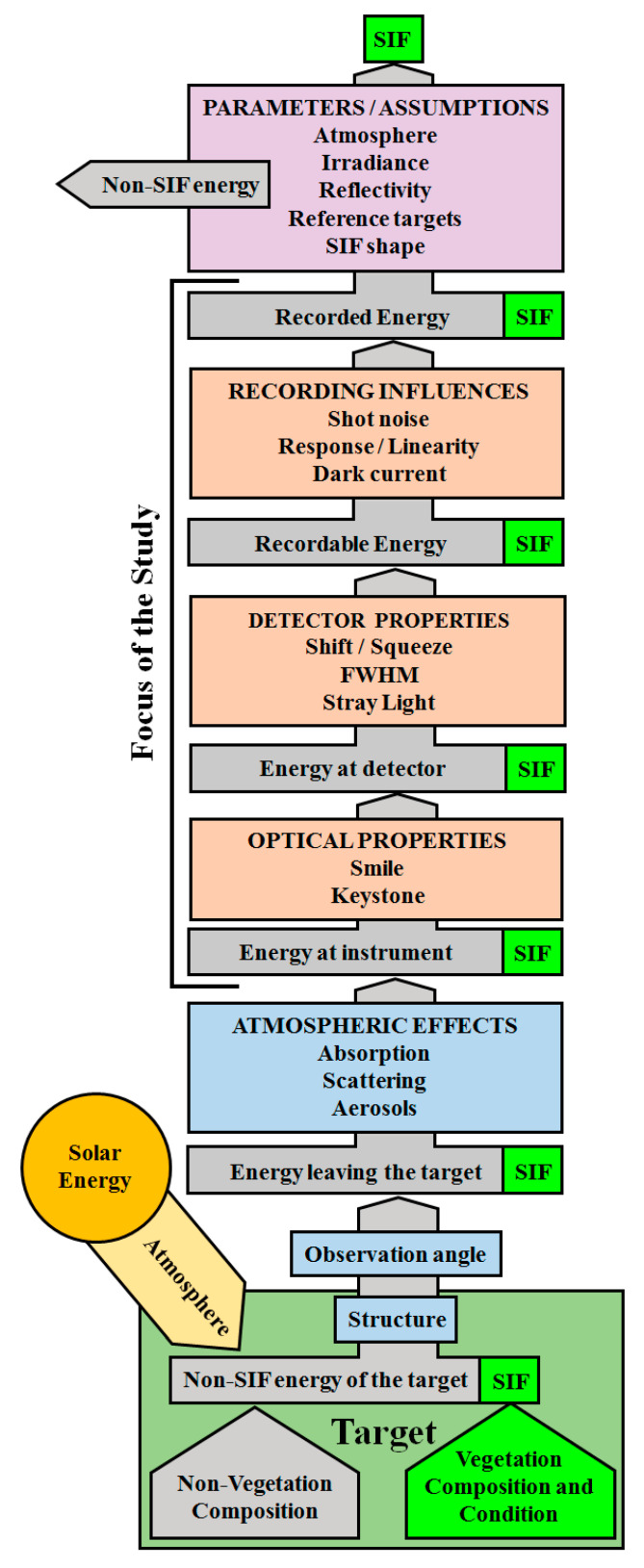
Flow-chart of potential influences on SIF signal. Influences in orange boxes are actively investigated in this study. Green components represent the SIF portion of the signal, and gray components represent the non-SIF portion of the signal.

Considering that SIF comprises such a relatively small amount of energy, it is important for the attributes and performance of SIF-retrieving instruments to be particularly robustly characterized. In pursuit of this standard, many studies have investigated the influence of sensor characteristics on the ability to retrieve SIF measurements. The full-width at half maximum (FWHM) [19], band spacing (spectral sampling resolution) [19], stray light [7,23], dark current [19], as well as instrument-specific sources of variation, such as non-linearity in detector responsivity [7], have all been shown to impact SIF estimations by masking the signal, or confounding the retrieval. Additionally, atmospheric conditions [24], aerosols, scattering and reabsorption [25], and bi-directional effects [26] have been shown to be important, particularly for more distant SIF retrievals from airborne and satellite platforms. Figure 2 summarizes the potential influences on SIF retrievals that need to be considered.

The methods to retrieve SIF from spectrometer observations have diversified in accordance with the requirements of particular instruments, acquisition conditions, and data product specifications [27]. Some retrieval methods, such as Fraunhofer line discrimination (FLD) and improved Fraunhofer line discrimination (iFLD), use a modelled or retrieved proportional radiance relationship between spectral bands to infer the additive presence of SIF [18]. Other retrieval methods attempt to separate the SIF component in one or more spectral bands, either by estimating and removing specific non-SIF components, or by removing the influence of non-SIF components as layers of systematic variation, with methods such as principle component analysis (PCA) or singular vector decomposition (SVD). Contemporary spectral fitting methods typically employ a combination of these approaches to consider the SIF signal amalgamated across many spectral bands and features [19,28,29,30], and as a result have shown considerable success in mitigating noise in well-established instruments.

The FIREFLY activity was a technology demonstration that flew as part of the G-LiHT system for two years of data acquisitions. NASA Goddard’s G-LiHT airborne imager provides a unique combination of lidar and optical remotely sensed data concerning ecosystem structure and function, collected simultaneously. It integrates commercial off-the-shelf lidar, hyperspectral, and thermal sensors in a compact, and portable system. The coincident data from the G-LiHT instrument package may address many challenges currently facing SIF retrievals. For example, lidar can provide observations of vegetation canopy structure, noted as essential for providing the link to leaf-level traits [4], and provide characterization of its influence on SIF [10], which has been determined to result in strong directionality of SIF [15,31]. The addition of a high resolution (~3 cm) Phase One optical camera can provide estimates of vegetation cover and type within FIREFLY footprints, providing important context to SIF retrievals [32,33].

This study aimed to provide the following: robust characterizations of the attributes of the FIREFLY sensor system relevant to SIF retrieval; assessments of various SIF retrieval methods in the context of these sensor system attributes; modelling tools, including the ability to simulate high resolution upwelling radiance spectra, to tailor design of future SIF products to specific sensor system attributes; and recommendations for further measurements and deployment strategies to optimize SIF retrieval.

## 2. Materials and Methods

Attributes of FIREFLY known prior to this study are displayed in Table 1. To quantify, or otherwise characterize, other attributes of FIREFLY that could be potential sources of uncertainty in SIF retrieval, several sources of data were utilized, each of which is described later, starting in Section 2.4. Firstly, however, information is provided about the conceptualization of SIF retrieval methods, SIF signals, and SIF retrieval error in this study, and their implementation in forward modelling and contextualization of results.

### 2.1. Representing SIF Retrieval Methods

The FIREFLY spectral range of 670–780 nm was designed to span the chlorophyll fluorescence emission spectrum (Figure 1), which encompasses both the oxygen A (~759–771 nm) and oxygen B (~688 nm) telluric atmospheric absorption features, and many solar Fraunhofer absorption features. This spectral range includes regions previously exploited for SIF retrievals, such as the “split window” either side of the oxygen A absorption feature (756–759 nm and 770.5–774.5 nm) [10]. As such, FIREFLY has access to many SIF retrieval approaches that differ in both their targeted spectral regions, and their fundamental mechanics. Since each SIF retrieval method considered will heavily contextualize the results, this study attempts to provide as wide a perspective as possible by utilizing two archetypes of SIF retrieval. Together, these two SIF retrieval archetypes approximate the mechanics of most major SIF retrieval methods, while remaining explicit in their propagation of uncertainty as deeply into their implementation as possible. The first of these archetypes is the “direct” retrieval, representing those methods that seek to remove non-SIF components of a signal, retrieving the residual of this process as the SIF signal. The second archetype is the “indirect” retrieval, representing methods that use baseline non-SIF relationships between spectral bands or regions to infer the presence and quantity of an additive SIF signal in collected observations.

Among contemporary methods that would be categorized here as “direct”, some strip away non-SIF components of the signal explicitly, such as retrievals from inside the oxygen features that estimate and remove attributes of solar irradiance, target reflectivity, and atmospheric effects. To be used operationally, the approach of retrieving SIF directly from oxygen features relies on an estimate of the non-SIF component of the radiance at the sensor, which could in turn involve estimates of some combination of the following, depending on the coincident data available: top-of-atmosphere irradiance, atmosphere from top to target, downwelling irradiance, target reflectivity, and atmosphere from target to sensor. While the potential to retrieve these components is discussed in Section 4.1.2, quantitative analyses of the uncertainty in this SIF retrieval method are focused on the expected contributions of noise, and other instrument sources.

Other direct methods remove non-SIF components implicitly, typically by identifying them as systematic variation via principal component analysis (PCA) or singular vector decomposition (SVD). Others still, including the prominent spectral fitting methods, use a combination of explicit knowledge of instrument and target function shapes, and the implicit structural properties of the signal, to derive a residual SIF function. Compared to the maturity and complexity of these approaches, the representations of direct retrievals in this study are simple, assuming that each band is used as an individual sample, with SIF and potentially confounding influences applied to it. While treating bands as independent samples may fall short of the potential performance of spectral fitting methods in fighting noise and removing systematic variation, this approach is employed here to provide transparent propagation of uncertainty, enabling independent evaluation of each potential source of uncertainty in SIF retrievals.

Indirect methods aim to utilize baseline knowledge of the quantitative relationship between the radiances of specific spectral bands in the absence of SIF (typically a proportion) to infer the presence of an additive signal of SIF in collected observations (see Appendix A.1 for the quantitative approach). When applied to Fraunhofer lines specifically, this approach is called Fraunhofer line discrimination (FLD), but the principle is the same when considering oxygen bands. The baseline knowledge of proportional depth can be established by utilizing a reference target that is known not to contain SIF, captured as part of an acquisition (an in-scene target), or a non-SIF-containing reference target that comes from a different acquisition or a simulation (an out-of-scene target).

**Figure 3:**
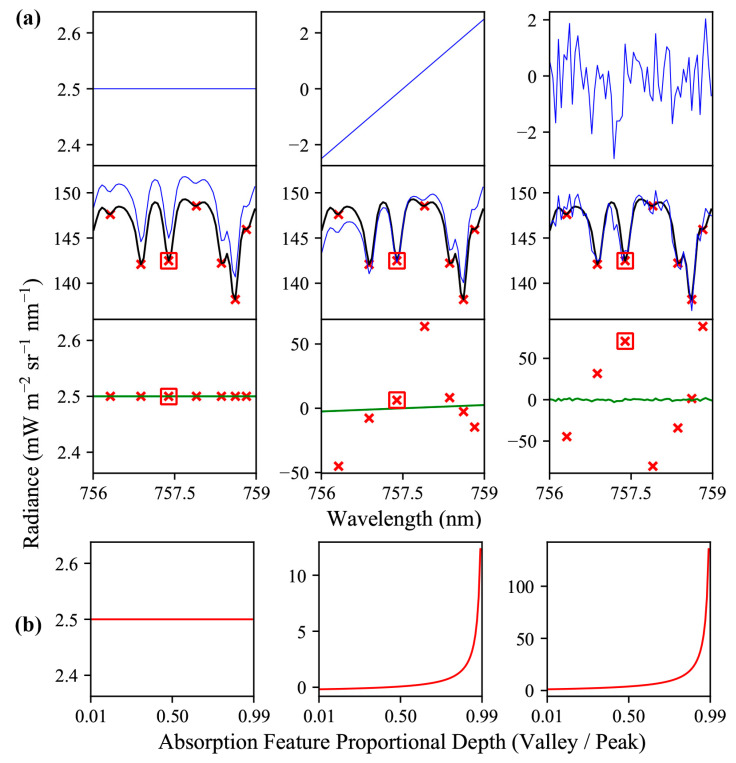
(**a**) Different types of influences (top row, blue line) that can confound SIF retrievals: uniform (left column), and non-uniform sloped (center column) and random (right column). Each of these influences is applied (middle row) to a sample of simulated FIREFLY spectra (black line), resulting in an adjusted spectrum (blue line). Valleys for indirect SIF retrieval (red x) are shown for seven absorption features. The resulting error in the SIF retrieval (bottom row) is shown for direct retrievals from every band (green line), and indirect retrievals from the absorption features (red x). (**b**) One absorption feature (identified by the red boxes in (**a**)) is simulated over a range of proportional depths to show the reaction of indirect SIF retrievals to uniform (left), sloped (middle), and random (right) influences.

By utilizing proportional relationships between band radiances, rather than relying on absolute radiance, indirect methods can circumvent the need to estimate, or otherwise account for, any influence that would scale the bands in question uniformly, such as atmospheric absorption, or target reflectivity. However, since indirect retrievals rely on pairs or groups of bands at different spectral locations, there can be some secondary reactions in the uncertainty in SIF retrievals when influences act non-uniformly on the targeted spectral bands. These principals are demonstrated in Figure 3. Indirect SIF retrieval methods presented in this study are based on pairs of bands for oxygen and solar absorption features, with each pair having an established proportional relationship between a lower-radiance “valley” band, and a higher radiance “peak” band. The processes of selecting the bands and quantifying the expected proportional radiance is detailed in Section 2.3.1.

### 2.2. Representing SIF Signals

Where a representation of a SIF signal was required, it was modelled based on the properties of the SIF signal displayed in Mohammed et al., 2019 [9]. The SIF signal was represented as the sum of two normal distributions representing photosystem II (with standard deviations of 10 nm, and 18 nm), and a further normal distribution representing photosystem I (with a standard deviation of 20 nm). The overall and relative scaling of the resulting SIF signal was controlled by three parameters: a value of SIF for the apex of the 740 nm peak; a scaling factor for photosystem I (0–1); and a scaling factor for photosystem II (0–1). For more details on the SIF simulation used here see Supplementary Materials and Appendix A.2, Table A1.

### 2.3. Representing FIREFLY Spectra

Whenever a representation of a noise-free FIREFLY spectra was required, it was convolved from a high-resolution (<0.001 nm) solar irradiance spectrum that included atmospheric influences [34], similar to those utilized by previous studies [9,35,36]. Each FIREFLY band was represented by a Gaussian resampling of the solar irradiance spectrum, with standard deviations derived from the FWHM function estimated by Goddard’s laser for absolute measurement of radiance (GLAMR) observations (as described in Section 3.1).

#### 2.3.1. Deriving Baselines for Indirect SIF Retrievals

To ascertain the locations and properties of absorption features that could potentially be used for indirect SIF retrieval, simulated spectra were derived for the full spectral range of each spatial pixel of FIREFLY, as described in Section 2.3. Each simulated spectrum (recorded at this stage in relative radiance units) was then searched for upward inflections. Upward inflections were then treated as the lower-radiance “valley” bands needed for indirect SIF retrievals (as described in Section 2.1), and each was paired with the “peak” band that would provide the deepest proportional depth within a search window of ±3 bands. During this process it was ensured that no valley shared a peak with another absorption feature.

#### 2.3.2. Forward Modelling with Simulated Spectra

A set of modelling tools, developed for this study, allowed the scaling of the FIREFLY simulated spectra to the radiances and reflective profiles of targets in real FIREFLY observations; the addition of artificial SIF signals; the addition of uniform, sloped, and random influences; and Monte Carlo simulation of any combination of these effects to investigate contributors to uncertainty in SIF retrievals. For more details about the modelling tools see Appendix A.3.

### 2.4. Characterizing FIREFLY with GLAMR

Goddard’s laser for absolute measurement of radiance (GLAMR, manufacturer, city, state, country) is a high-quality calibration facility, utilized by this study for several purposes: to characterize detector response and dark current; to estimate FWHM and signal to noise ratio (SNR) for each of the 266 spatial pixels of FIREFLY; and to assess spectral stray light. GLAMR is NASA Goddard Space Flight Center’s portable version of the National Institute of Standards and Technology (NIST) Spectral Irradiance and Radiance responsivity Calibrations using Universal Sources (SIRCUS) [37]. It consists of a wavelength-tunable laser coupled to an integrating sphere to produce a near-monochromatic source of uniform radiance. It facilitates a full aperture absolute calibration approach and a spectral scan of an instrument’s operating spectrum allows determination of the absolute spectral responsivity (ASR) of an instrument for all of its spectral channels [38] (Figure 2). GLAMR is designed to provide uncertainty of a sensor’s radiance responsivity to around 0.5 to 5% depending on spectral region. Radiance provided by the GLAMR system is traceable to the primary standards at NIST. The international system of units (SI) traceability to the NIST’s primary optical watt radiometer (POWR) is established using transfer radiometers [39,40].

The GLAMR facility was used to characterize the response of FIREFLY in wavelengths ranging from 690 nm to 1000 nm (Table 2). Between the wavelengths of 690 nm and 764 nm, four wavelength ranges were sampled at 0.05-nm steps, and six ranges were sampled at a lower resolution of 0.15 nm. The full wavelength range of 690 nm to 1000 nm was sampled at 1-nm steps to investigate spectral stray light from within and beyond the spectral range of the sensor. Background (laser source is shuttered closed) data were collected for about 10 seconds after every 10 seconds of open shutter.

**Figure 4:**
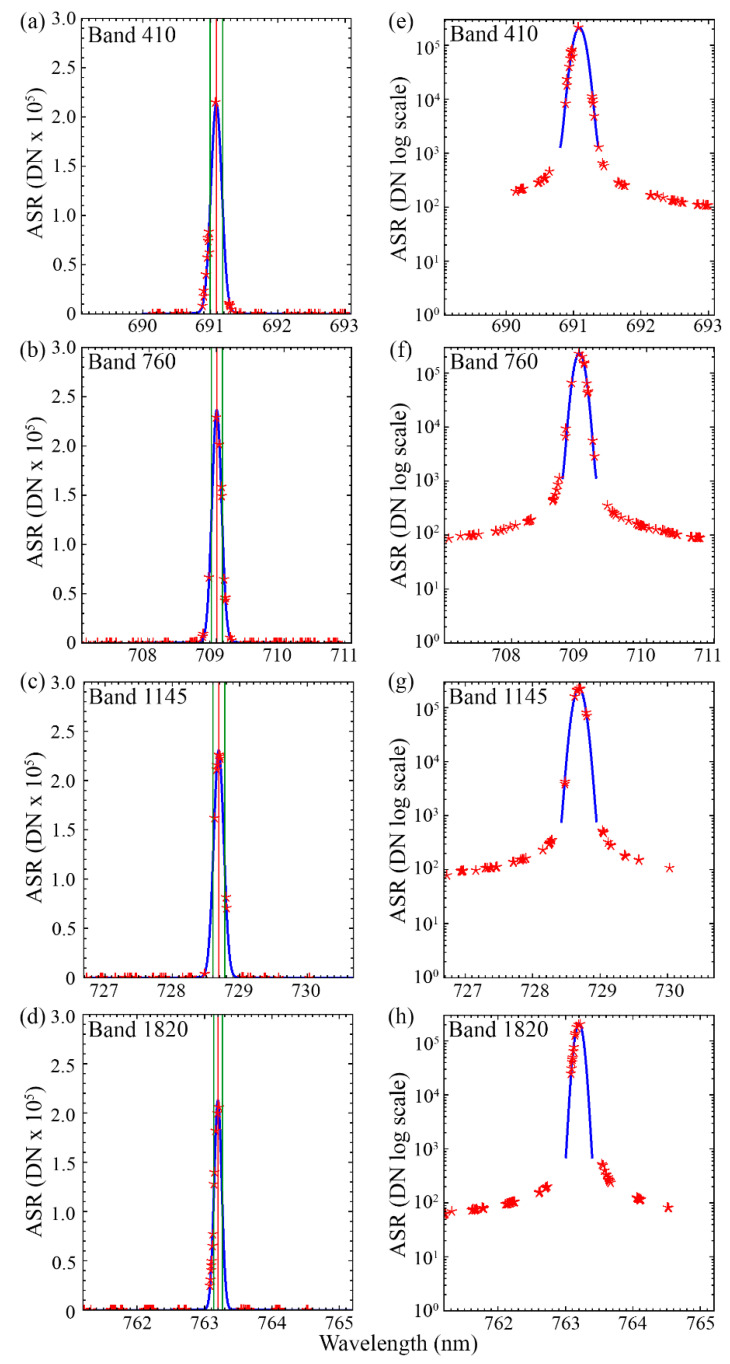
Examples of absolute spectral response (ASR) functions for several FIREFLY bands. The actual observations (red stars) are interpolated with a Gaussian function (blue line) to calculate the full-width at half maximum (FWHM) (green lines) for the band center (red line). The full observations are shown in panels (**a**–**d**) for the band numbers stated in the panels. The fit of the function to the peak of the observations for the same bands are shown in panels (**e**–**h**).

The sensor response digital numbers (DN) were then matched to corresponding data from GLAMR using synchronized time stamps. GLAMR integrating sphere radiance measurements were combined with the background adjusted DNs, to provide absolute spectral response (ASR) for each spectral pixel [41] (Figure 4). GLAMR radiance measurements with high wavelength variance (standard deviation > 0.03) were excluded from the study. All of the ASRs were visually inspected, and those with insufficiently sampled central peaks were excluded. The ASRs were then interpolated to a wavelength step of 0.001 nm to facilitate characterization of reported bandwidths of <0.2 nm. A Gaussian fit was used to interpolate the central peak area. After the described filtering had been implemented, 104 bands were sufficiently characterized.

After interpolation of ASR to a uniform wavelength step, the full-width at half maximum (FWHM), was estimated using the following two equations:

(1) $$Band\;Responsivity\;=\;\overset{\lambda =1000}{\underset{\lambda =690}{\sum }}ASR\left(\lambda \right)\cdot \Delta \lambda$$

(2) $$FWHM\;=\frac{1}{ASR_{MAX}}\;\overset{\lambda =1000}{\underset{\lambda =690}{\sum }}ASR\left(\lambda \right)\cdot \Delta \lambda =\frac{Band\;Responsivity}{ASR_{MAX}}$$

where Δλ is wavelength spacing (0.001 nm) between interpolated measurements.

There are two kinds of stray light considered here, spectral and spatial. Spectral stray light is described by the spectral stray light signal distribution function (SDF) and spatial stray light is described by the point spread function (PSF). Here we have focused on characterizing spectral stray light, since spectral stray light is potentially highly influential on SIF retrievals. Stray light is mostly caused by non-ideal behavior of the optical components (mirrors, slits, and grating etc.).

### 2.5. Characterizing FIREFLY with Integrating Sphere

A Helios D Series uniform source integrating sphere configured with 150 W tungsten lamp and variable attenuator (Labsphere, North Sutton, NH) was used to observe FIREFLY response over a series of radiance levels from 0 to ~1300 mW. Sphere irradiance was also recorded with an ASD spectrometer to provide calibration measurements for FIREFLY response. To ensure an accurate dark current correction for the observations, dark current was measured in every band over five repeats and then averaged.

### 2.6. Characterizing FIREFLY with Controlled Acquisitions

An additional series of observations were made with FIREFLY mounted on a bench, outside, in full sunlight around solar noon, pointed downward at a consistent angle of approximately 30° below horizontal. Four targets were placed, in turn, at a consistent distance of approximately 0.5 m from the instrument ensuring that exclusively the target was observed by the full cross-track swath of FIREFLY. Three Lambertian panels were used as targets: white, gray (20% reflectivity), and black (5% reflectivity). Additionally, a fourth target consisting of spinach leaves was utilized. The leaves were arranged to provide ubiquitous, flat coverage on a tray. For each target, 10,000 consecutive observations were made before replacing it with the next target. The full set of targets was observed three times, for a total of 30,000 observations per target. The first set of observations for each target were later found to be contaminated by shadowing and were therefore removed from consideration. The second and third sets of observations for each panel were examined, and sections with upward or downward trends over time were removed, as these represented a change in irradiance conditions.

### 2.7. Characterizing FIREFLY with In-Flight Acquisitions

A summary of in-flight acquisitions used in this study can be found in Appendix A Table A2. These observations were collected opportunistically during various deployments with FIREFLY mounted on an aircraft as part of the G-LiHT instrument package. Typically, G-LiHT flies at an altitude of 335 m, giving FIREFLY a swath width of 140 m, unless otherwise noted. The in-flight data is mostly analyzed and presented without applying orthorectification to ensure that the images can interpreted according to their direct relationship with the FIREFLY detector array.

## 3. Results

### 3.1. Spectral Channel FWHM

**Figure 5:**
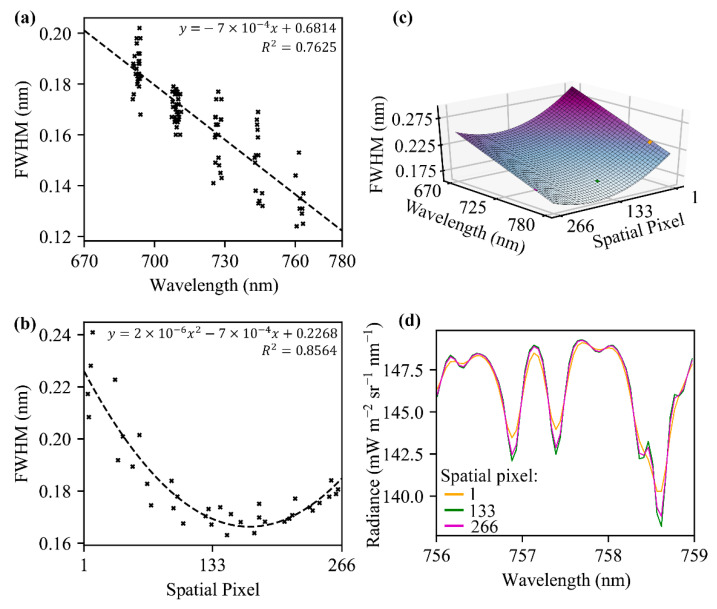
(**a**) The relationship (black dashed line) between FIREFLY FWHM and wavelength for spatial pixel 133, based on observations made with GLAMR (black x); (**b**) the relationship (black dashed line) between FIREFLY FWHM and spatial pixel for spectral band 922 (717.11 nm), based on observations made with the integrating sphere and ASD spectrometer (black x); (**c**) surface showing the combined FIREFLY FWHM function for spatial pixel and wavelength; and (**d**) examples of sections of spectra (colored lines) drawn from different spatial pixels (represented by same colored patches in (**c**)).

According to the observations made with GLAMR, the FWHM of FIREFLY spectral channels varied between ~0.12 nm to ~0.2 nm, and a negative relationship was observed between FWHM and wavelength (Figure 4). Further investigation in the spatial dimension of the sensor showed the FWHM is also spatially dependent (Figure 5). The combined FIREFLY FWHM was fitted as an exponential relationship with spatial pixel and wavelength:

(3) $$FWHM=2\times 10^{-6}D-7\times 10^{-4}D-7\times 10^{-4}W+0.76405$$

where *D* is the spatial pixel number of FIREFLY (1–266) and *W* is the wavelength in nanometers of the spectral band of FIREFLY. The standard deviation of the residual of this fit was 7.32 × 10^−3^ nm.

### 3.2. Band Centers

**Figure 6:**
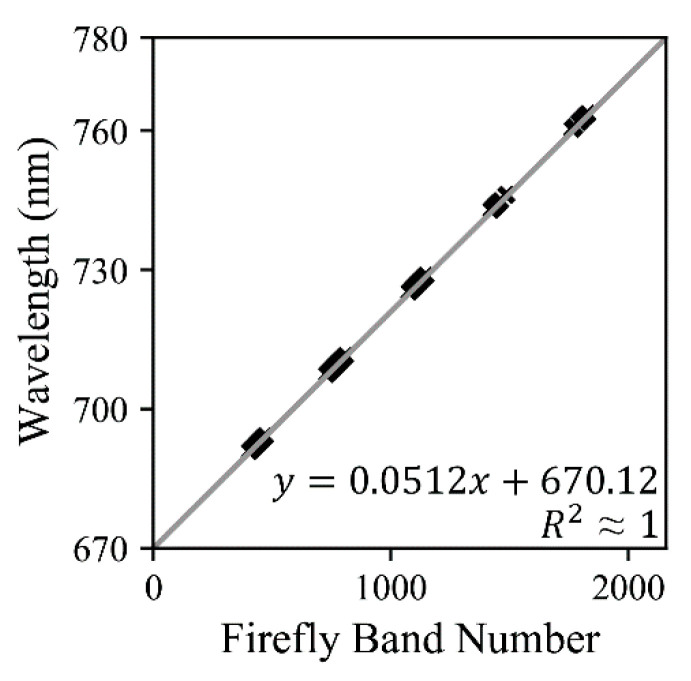
The relationship between FIREFLY band number and wavelengths (gray line) as established with GLAMR observations (black x).

The band numbers of FIREFLY were found to have a near-perfect linear relationship with wavelength (R^2^ ≈ 1) (Figure 6):

(4) $$W=5.12\times 10^{-2}\cdot B+670.12$$

where *W* is the wavelength in nanometers and *B* is the band number (1–2166).

### 3.3. Detector Response and Linearity

**Figure 7:**
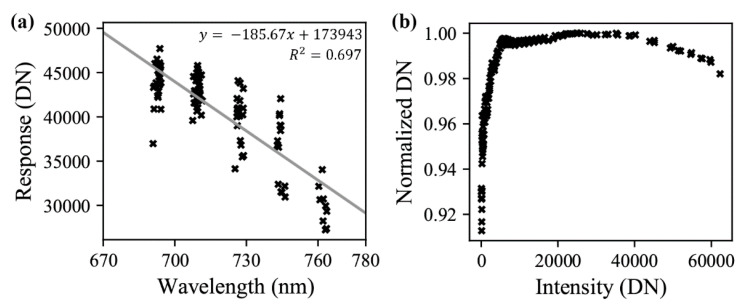
(**a**) The relationship between FIREFLY response and wavelength (gray line), established by GLAMR observations (black x); (**b**) the relationship between normalized digital numbers (DN) and intensity DN used to establish the detector non-linearity, as measured with the GLMR integrating sphere monitors and ASD (black x).

The detector response across the spectral range of FIREFLY bands (Figure 7) was measured in DN W^−1^ m^−2^ sr^−1^ nm^−1^ as

(5) R=−185.67·W+173943

where *R* is the band response in digital numbers (DN) and *W* is the wavelength in nanometers. 

The detector non-linearity was assessed (Figure 7b) and found to be linear to within 0.3% for 10,000–50,000 DN, where most retrievals would reside. However, the non-linearity correction derived from GLAMR has since been replaced by a correction provided by the manufacturer. Since this replacement correction is based on observations at 32 points on the spatial array of FIREFLY, it is believed to be more precise than the correction derived from the observations in Figure 7.

### 3.4. Dark Current

**Figure 8:**
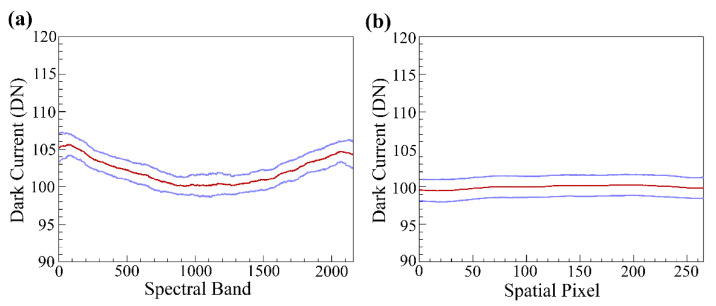
(**a**) Mean dark current (red line) as measured with GLAMR observations. Additionally, shows ±1 standard deviation (blue lines) across FIREFLY spectral bands; (**b**) mean dark current (red line) as measured with GLAMR observations. Additionally, shows ± 1 standard deviation (blue lines) across FIREFLY spatial pixels.

Dark current was investigated across both the spectral and spatial dimensions (Figure 8). Dark current across the spatial domain was observed to be quite stable with no systematic trends. However, across the spectral domain there was a clear “smile” observed, with dark DN of 100 at center, and 105 DN at each end. Dark DNs of pixels at both corners of the spectral domain were significantly higher than the rest at about 115. The standard deviation of dark current variability was mostly lower than 2 DN throughout the sensor array.

### 3.5. Shot Noise

**Figure 9:**
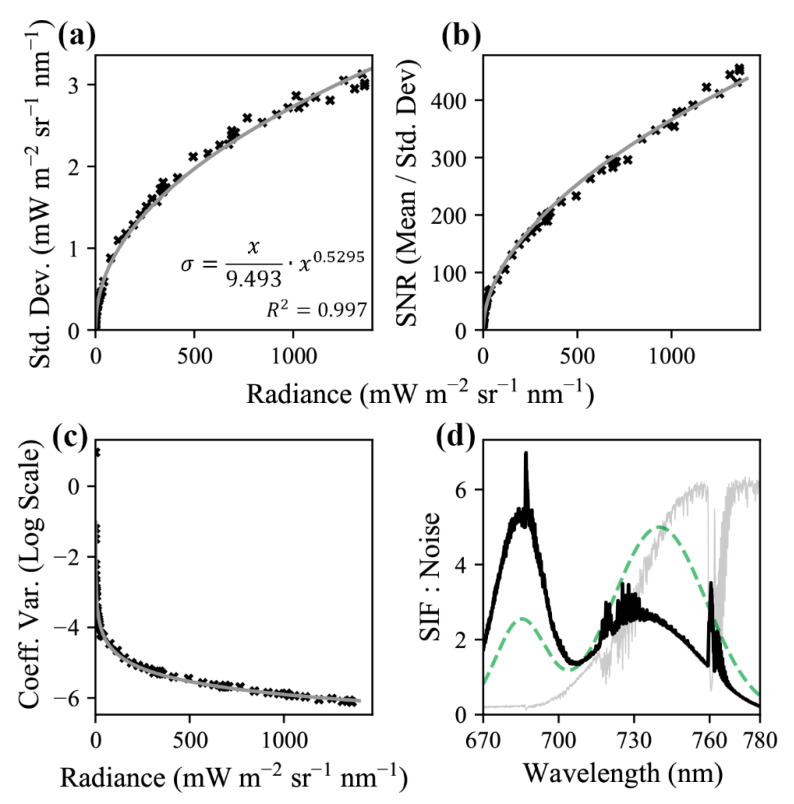
(**a**) The relationship (gray line) between FIREFLY radiance and standard deviation based on observations made with the integrating sphere and ASD (black x); (**b**) the relationship (gray line) between the signal to noise ratio (SNR) and radiance, based on observations made with the integrating sphere and ASD (black x); (**c**) relationship (gray line) between coefficient of variation and radiance based on sphere observations (black x); and (**d**) an example FIREFLY vegetation spectra (gray line) with an example SIF signal (green dashed line, not drawn to scale) and the ratio of SIF to the expected noise based on the radiance values from the example vegetation spectra (black line).

For many spectroscopy applications, noise is expressed as a ratio of instrument radiance to the expected variation at that radiance (a signal to noise ratio). However, for SIF retrievals, the most important relationship is between the noise and the expected magnitude of SIF, since SIF does not reliably co-vary with radiance. Therefore, noise results are most usefully expressed as absolute values of noise (Figure 9). The highly conserved relationship (R^2^ = 0.997) of the expected standard deviation of the noise with radiance was found to be

(6) $$\sigma =365.99\cdot R^{0.5295}$$

where *σ* is the expected standard deviation in mW m^−2^ sr^−1^ nm^−1^ and *R* is the radiance of the FIREFLY signal, also in mW m^−2^ sr^−1^ nm^−1^.

**Figure 10:**
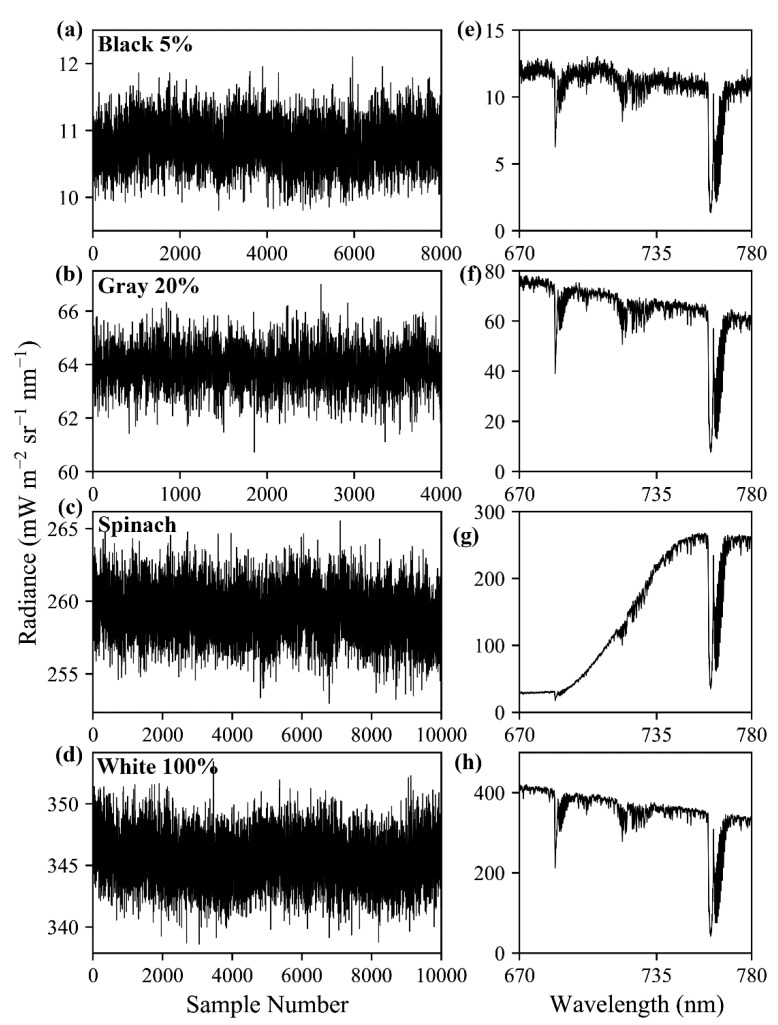
The radiance of FIREFLY spatial pixel 133, band 1272 (735.003 nm) over thousands of consecutive samples (**a**–**d**) of four targets, with an example of a single spectrum (**e**–**h**) from each panel, taken from spatial pixel 133.

**Figure 11:**
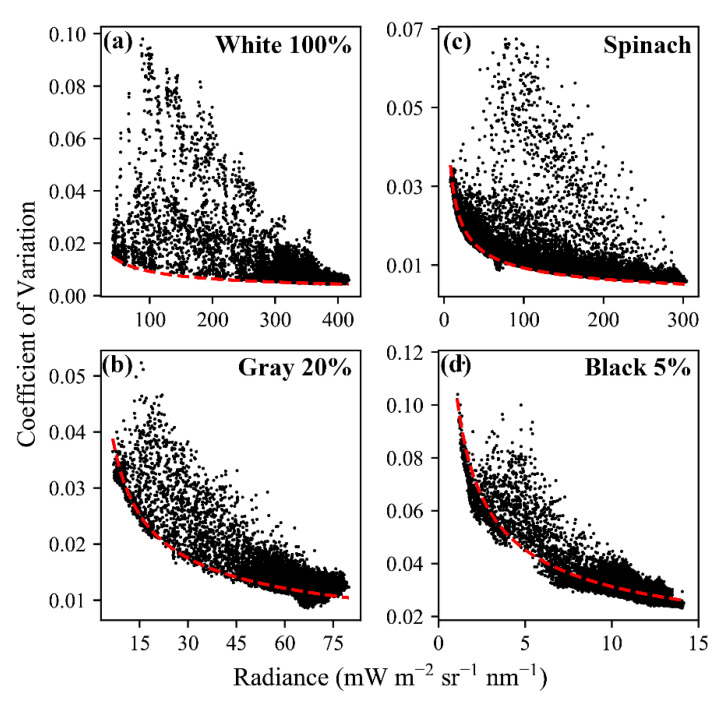
The coefficient of variation of FIREFLY radiance in spatial pixel 133, all bands, over thousands of consecutive samples (black dots) of four targets; white (**a**), gray (**b**), spinach (**c**) and black (**d**); compared to the expected coefficient of variation based on the noise characterization with the integrating sphere and ASD (red dashed line).

**Figure 12:**
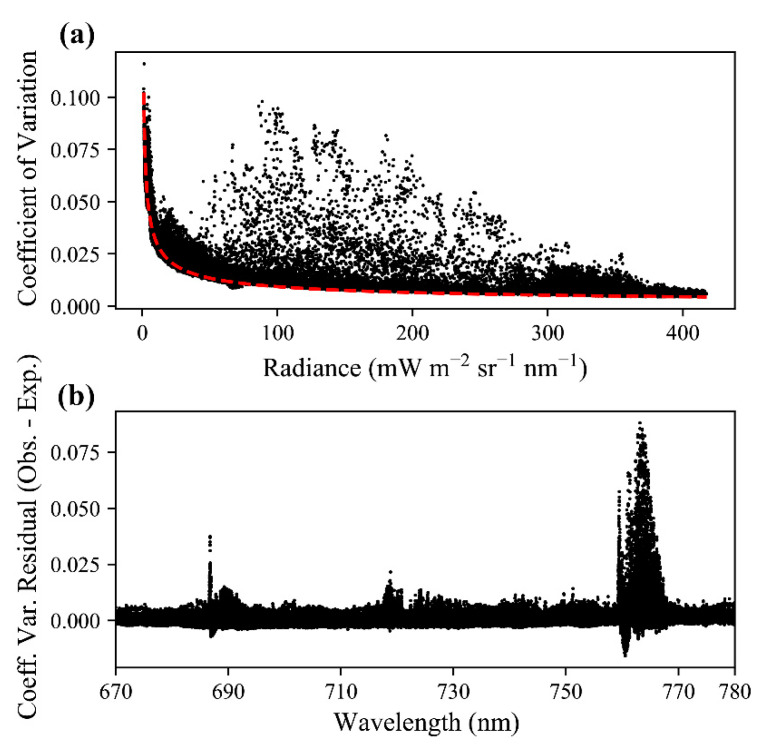
(**a**) The coefficient of variation of FIREFLY radiance in spatial pixel 133, all bands, all targets over thousands of consecutive samples (black dots), compared to the expected coefficient of variation based on the noise characterization with the integrating sphere (red dashed line). (**b**) The residual of the coefficient of variation (observed–expected) (black dots) based on the samples in (**a**).

Results from the controlled acquisitions of thousands of observations of four targets (white, gray, black, and spinach) (Figure 10), as described in Section 2.6, exhibited higher magnitude variation in the radiance of FIREFLY bands than would be expected based on the characterization of noise with the integrating sphere (Figure 11). The residuals between the expected coefficients of variation and the observed coefficients of variation were generally negatively associated with radiance (Figure 12). In other words, the variation observed agreed more closely with the expected variation from noise as the radiance increased.

### 3.6. Stray Light

**Figure 13:**
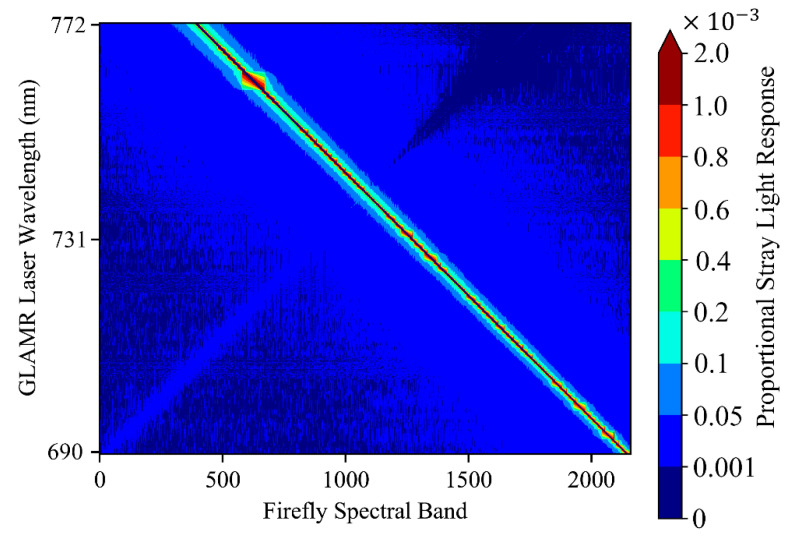
The proportional response of FIREFLY bands to a range of wavelengths of light emitted by the GLAMR laser. Note the non-linear, categorical color-ramp.

FIREFLY demonstrated very little influence of stray light across its spectral range (Figure 13), suggesting the characterization of FWHM by estimation of ASR (Figure 4) was appropriate. Investigation of far-field stray light in wavelengths ranging from 800 nm to 1000 nm found that the FIREFLY sensor response was quite similar to dark current, with dark current adjusted DN remaining close to zero, with a standard deviation of 0.1 DN.

### 3.7. Forward Modelling

**Figure 14:**
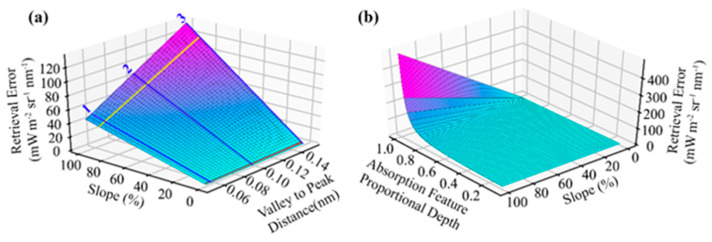
(**a**) Modelled relationship between error in an indirect SIF retrieval, the gradient of a sloped influence, and the spectral distance between a valley and a peak for an absorption feature of 0.9 proportional depth. For context, the approximate steepness of the leaf reflectivity (yellow line), and the SIF function (red line) at 730 nm, based on Figure A, are shown. Spectral distance equivalent to 1, 2, and 3 band intervals of FIREFLY (blue lines) are shown. (**b**) Modelled relationship between error in an indirect SIF retrieval, the gradient of a sloped influence, and the proportional depth of an absorption feature.

**Figure 15:**
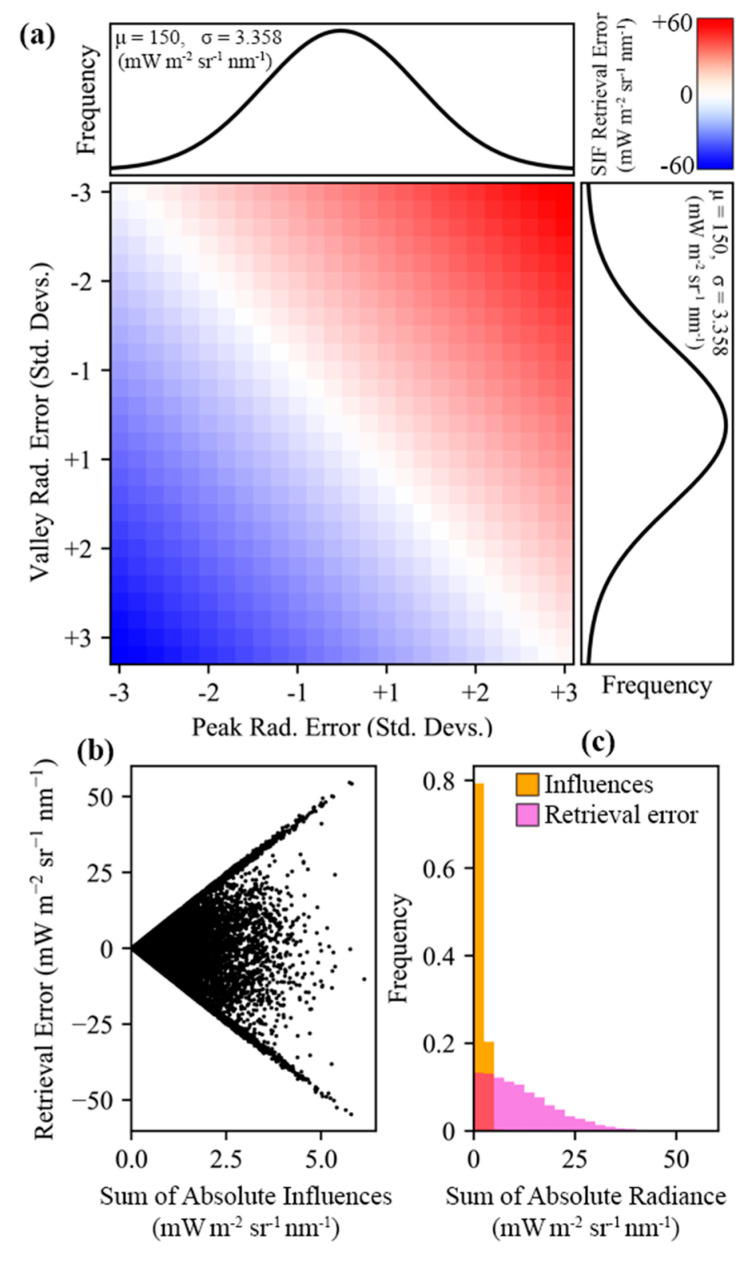
(**a**) Monte Carlo simulation of 10,000 pairs of random influences applied to a peak and a valley for indirect SIF retrieval, drawn from normal distributions (black lines) based on measured FIREFLY shot noise; (**b**) SIF retrieval error plotted against the sum of absolute influences applied to the valley and peak bands (black dots); and (**c**) distributions of sum of absolute influences and resulting errors in indirect SIF retrieval.

The results from the characterization of FIREFLY with GLAMR, the integrating sphere, controlled acquisitions, and in-flight data were used to parameterize various forward models that related instrument specifications, and other potential sources of uncertainty, to downstream error in SIF retrievals. For the most part, these modelling efforts were conducted to contextualize specific discussion points, and their outcomes can be found in the subsequent sections. However, some general relationships between the types of sources of uncertainty and SIF retrieval error that were prevalent in the modelling efforts can be demonstrated here. These relationships are mostly intuitive, given the principles of SIF retrieval outlined in the method, and can be summarized as follows:

1. Uniform influences on spectra intended for SIF retrieval will result in one-to-one error in both direct and indirect SIF retrievals (Figure 3).
2. Non-uniform sloped influences produce one-to-one error in direct retrievals (Figure 3).
3. All non-uniform influences produce amplified errors in indirect SIF retrievals (the magnitude of the error is higher than the sum of the influences on the relevant bands) (Figure 3).
4. Sloped influences produce positive errors in indirect SIF retrievals if the direction of the slope increases the valley radiance relative to the peak radiance, and negative errors if the peak is increased relative to the valley.
5. Sloped influences produce higher magnitude errors in indirect SIF retrievals when there is a steeper slope; when the spectral distance between the valley band and the peak band is greater; or when the absorption feature is shallower (the proportional depth is higher) (Figure 14).
6. Random influences produce one-to-one error in each band used for direct retrievals (Figure 3).
7. Random influences that are expected to be normally distributed (such as shot noise) produce bimodal error distributions in indirect SIF retrievals (Figure 15).

## 4. Discussion

FIREFLY was designed to cover a spectral range that would facilitate SIF retrievals via direct or indirect methods, and utilizing oxygen absorption features, solar absorption features, or spectral fitting. For each SIF retrieval approach, different attributes of the FIREFLY instrument hold critical importance, and there are different requirements for those attributes. Some attributes need to meet a particular standard, whereas other simply need to be characterized to a particular standard. Additionally, each SIF retrieval approach requires different supporting information, whether from coincident measurements or modelling; requires different assumptions to be met; and begets different, and sometimes even conflicting, recommendations for retrieval conditions and data processing techniques. The subsections of Section 4.1 each explore a different potential SIF retrieval approach for an operational FIREFLY product, evaluating the specifications and characterizations of FIREFLY in the context of the retrieval approach, and identifying the assumptions, supporting information, and further studies that would be required to enact the approach.

### 4.1. SIF Retrievals with FIREFLY

An important consideration for the discussions that follow is that the observed variation in radiances in the controlled acquisitions, which were conducted outside, exceeded the expected variation (see Section 3.5, and Figure 10, Figure 11 and Figure 12), in some cases by a large amount. The expected variation was based on the characterization of shot noise with the integrating sphere, which appeared to suggest a stable and highly conserved relationship (R^2^ = 0.997) between shot noise and radiance (Figure 9). There are many potential explanations for why the observed noise was higher than expected in the controlled acquisitions, including the possibility that it is a more accurate representation of the noise that should be expected in operational FIREFLY retrievals. However, it seems more likely that the results were influenced by accidental shadowing from clouds or branches; heating of the instrument in direct sunlight; scattering or reflections from objects near the instrument; or vibration of the mounting surface perturbing the spatial relationship of the instrument and the targets. Since there are several potential, and entirely plausible, confounding variables associated with the controlled acquisitions, the following discussions are held in the context of the noise characterization from the integrating sphere. Regardless, investigating operational noise for FIREFLY through additional and refined controlled acquisitions is the highest priority for future studies.

#### 4.1.1. Indirect Retrievals from Solar and Oxygen Absorption Features

Indirect SIF retrievals are those that utilize baseline knowledge of the relative radiance expected between a pair, or two groups, of bands in the absence of SIF to detect the presence, and quantify the magnitude, of an additive SIF signal. The most common form this approach takes, is to estimate the proportional depth of an absorption feature that is expected to be consistently represented in instrument observations. The main advantage of indirect retrievals is that there is no requirement for any estimation of irradiance conditions, nor of target reflectance, nor of any other influence that would scale the bands equally.

**Figure 16:**
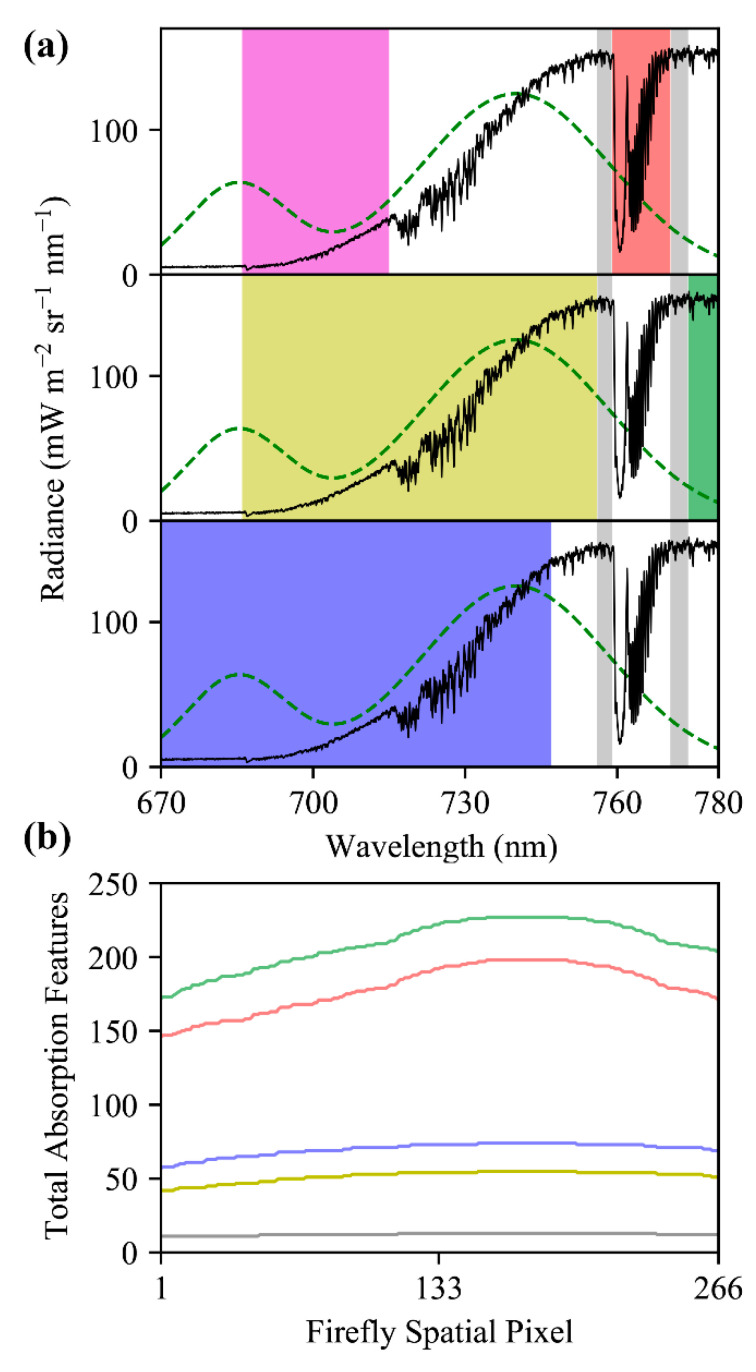
(**a**) An example FIREFLY vegetation spectra (black line) with a simulated SIF signal (green dashed line, not to scale). Each panel highlights a spectral region that might require different assumptions or supporting information: The top panel shows oxygen B (pink) and oxygen A (red); the middle panel shows sloped leaf reflectance (yellow) and the region where SIF can be expected to be polynomial in shape (green); the bottom panel shows the region subject to the influence of water vapor (blue); and all panels show the narrow regions (gray) not included in any other spectral region. (**b**) The number of solar absorption features found across the spatial dimension of FIREFLY, if each spectral region is excluded (line colors correspond to regions in (**a**), with red incorporating both oxygen A and oxygen B regions).

The first consideration for attempting indirect SIF retrievals is the availability and properties of the absorption features represented in the acquisitions of an instrument. As described in Section 2.3, absorption feature valleys were identified in simulated FIREFLY spectra for the full spectral range of each spatial pixel, and then paired with spectrally proximal peak bands. The detailed characterization of FWHM in this study was essential to this process, as the expected depth of absorption features changes with the FWHM, and some absorption features even disappear entirely across the FWHM range of FIREFLY. Figure 5 shows examples of absorption feature depth varying with FWHM, and Figure 16 shows the variation in the number of absorption features represented in each FIREFLY spatial pixel.

**Figure 17:**
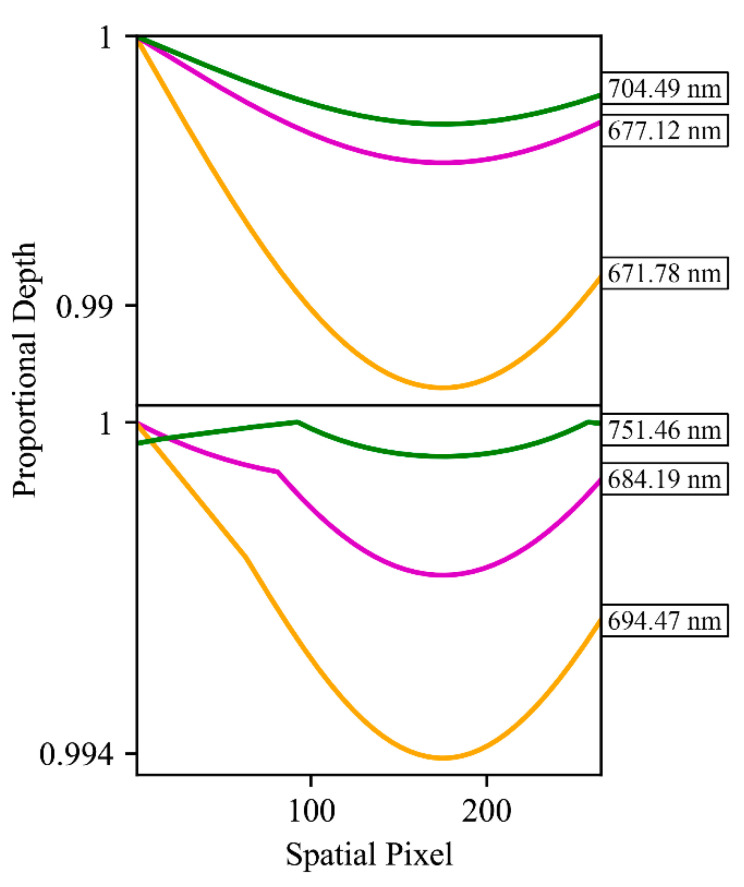
Examples of absorption feature proportional depths (colored lines and valley wavelength labelled) changing over the spatial pixels of FIREFLY as a result of the variation in FWHM.

The importance of characterizing FWHM thoroughly, and simulating absorption features accurately, is further emphasized by Figure 17. This figure shows that the proportional depths of absorption features change non-linearly with FWHM across the spatial pixels of FIREFLY. Furthermore, there are some examples of sharp inflections in the proportional depths, resulting from the changing FWHM causing a band to gain or lose the influence of prominent shapes in the underlying high-resolution reference spectra from which the simulated FIREFLY spectra are convolved. Because of these factors, if an instrument has FWHM variation, then it is essential that the baseline knowledge of feature proportional depths for indirect retrievals considers each spatial pixel separately, as is achieved here. Even if a real reference target is used, it must be observed separately with each spatial pixel of the instrument, which could be challenging for in-scene reference targets. For in-scene reference targets, the recommendation would be to pre-identify a ground target that could provide relatively homogenous cover across the FIREFLY swath when the instrument is at its typical acquisition altitude. Examples of suitable targets could include a perpendicular runway, empty highway, calm body of water, or large roof.

To quantify the importance of the thorough characterization of the FWHM variation of FIREFLY, simulated reference spectra, based on the lowest FWHM in the detector array (0.16 nm), was used as the baseline knowledge of proportional depth for a SIF retrieval from all spatial pixels, across all the in-flight FIREFLY data in this study. This approach provides an approximate integration of the error that would result in SIF retrievals if the minimum FWHM observed for FIREFLY was assumed for all spectral bands and spatial pixels. Targets that should not have contained SIF, determined by normalized difference vegetation index (NDVI) value of 0, had a mean absolute SIF estimate equivalent to 4.3% of target radiance in each band (±2.9, 95% CI), which was also equivalent to 161% of expected SIF, based on the example simulated SIF function shown in Figure 1 (±51.2, 95% CI). This would obviously be an unacceptable level of uncertainty, and confirms that a robust FWHM characterization is essential for FIREFLY to attempt indirect SIF retrievals.

The next step towards an indirect retrieval from FIREFLY would be to choose an appropriate subset of the available absorption features from which to estimate SIF. Several factors affect the choice of whether to include a particular absorption feature: the spectral region in which it resides; its proportional depth; the expected radiance, and therefore the expected noise, of its valley and peak bands; and the expected relative magnitude of SIF at its spectral location. It should be noted that these factors are far from independent, and that the selection of appropriate thresholds to make a decision based on each of these factors is likely to be a complex and dynamic process for an operational SIF retrieval product. However, establishing the tools for making such decisions for FIREFLY retrievals was among the aims of this study, and these are demonstrated below.

**Figure 18:**
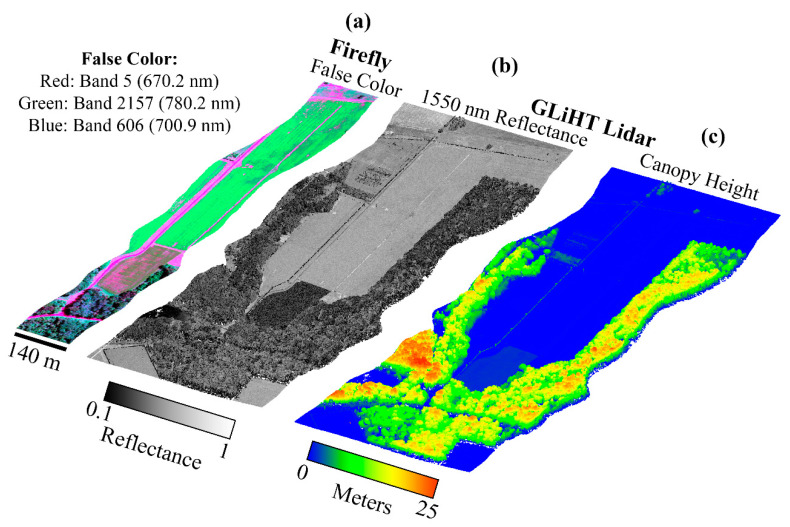
(**a**) Orthorectified false color RGB FIREFLY image (red: 670.2 nm, green: 780.2 nm, and blue: 700.9 nm) of an in-flight acquisition of agricultural land and woodland in Maryland, United States (greens: vegetated targets; purples/pinks: non-vegetated targets); (**b**) lidar point cloud, colored by intensity. Note the wider swath width of G-LiHT lidar instruments; and (**c**) lidar point cloud, colored by height above ground.

Several regions of the spectral range of FIREFLY (670 nm–780 nm) would require particular assumptions to be met, or additional information to be acquired in order to effectively utilize absorption features in those regions (Figure 16). The regions containing the oxygen A (759 nm–770.5 nm) and oxygen B (686 nm–715 nm) features are subject to the influence of oxygen absorption, and therefore would require atmospheric conditions to be accounted for in each in-flight collection. Likewise, the region influenced by water vapor (< 747 nm) would need a quantitative representation of atmospheric conditions. Atmospheric modelling for each in-flight acquisition would be the most likely solution for these regions, to which coincident data from other G-LiHT instruments could prove useful. For example, the downwelling radiometer could provide irradiance profiles, and the lidar instruments could provide ranges to estimate the length of the atmospheric column for each ground target (Figure 18). Ultimately, any errors in characterizations of atmosphere would propagate to error in SIF retrievals in these regions. Since even minor sloped influences have been shown to produce relatively large errors in SIF retrievals (Figure 3 and Figure 14), further study into an appropriate atmospheric model, and the accurate retrieval of parameters with G-LiHT data would have to be conducted before these regions could be used in an operational product.

**Figure 19:**
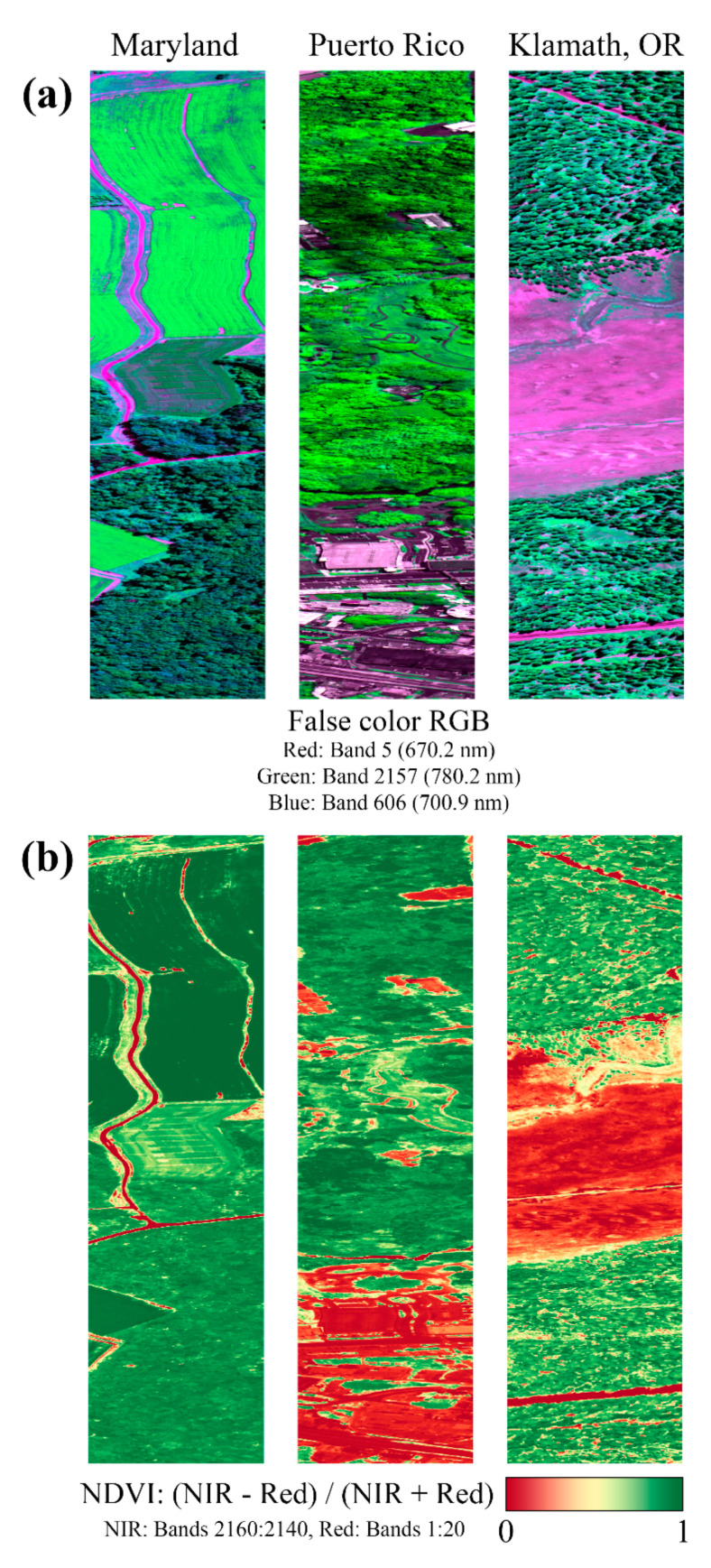
(**a**) False color RGB FIREFLY images (greens: vegetated targets; purples/pinks: non-vegetated targets); (**b**) NDVI FIREFLY images. Note that NDVI successfully removes the cloud shadows and shading from structure visible in the false color images.

Leaf reflectance is expected to have a steep slope in the central region of FIREFLY (756 nm–686 nm). To utilize absorption features in this region effectively, an estimate of the slope would be required. Leaf reflectivity slopes are well-represented in FIREFLY data (Figure 1 and Figure 10), and could be estimated with a ratio of bands similar to the process of NDVI (Figure 19). Using groups of bands for this process could help to mitigate the effects of noise and reduce uncertainty, which is particularly important given the sensitivity of indirect SIF retrievals to sloped influences (Figure 3). It should be noted that a substantial portion of the region with leaf reflectance shape overlaps with the region of water vapor influence, and the region of oxygen influence around the oxygen B feature. Furthermore, the remaining portion of the leaf reflectance region that is expected to be free of the atmospheric influences is also expected to have a more complex reflectance shape (Figure 16). These sorts of confounding interactions between influences are one of the main motivations behind direct SIF retrieval techniques such as PCA and SVD that remove observably systematic variation without attributing it to a specific origin. However, these techniques cannot be applied to indirect retrievals, and therefore this region will be challenging to use in an operational product.

Finally, the spectral region of FIREFLY acquisitions in which SIF is expected to have a polynomial shape (>774 nm) could experience some additional uncertainty as a result of the slope varying across relatively short spectral distances. Furthermore, this nuanced influence of the SIF shape in this narrow spectral region serves to highlight the wider challenge of using indirect SIF estimates to derive a shape for SIF, considering the valley and peaks bands for each absorption feature will be influenced non-uniformly according to the local SIF shape (Figure 3). As demonstrated in Figure 14, reducing the spectral distance between valley bands and peak bands can help mitigate violations of the assumption that energy in the bands will be scaled equally. However, this is balanced against the consideration that selecting pairs of bands that result in an increase in the depth of the absorption feature also mitigates non-uniform influences. In this study, a search window of ±3 bands (±0.153 nm) around a valley was used to select the peak that would provide the greatest depth of absorption feature. While this approach was chosen to balance the benefits of absorption feature depth and spectral proximity, further study is required to find optimum thresholds for absorption feature band selection.

**Figure 20:**
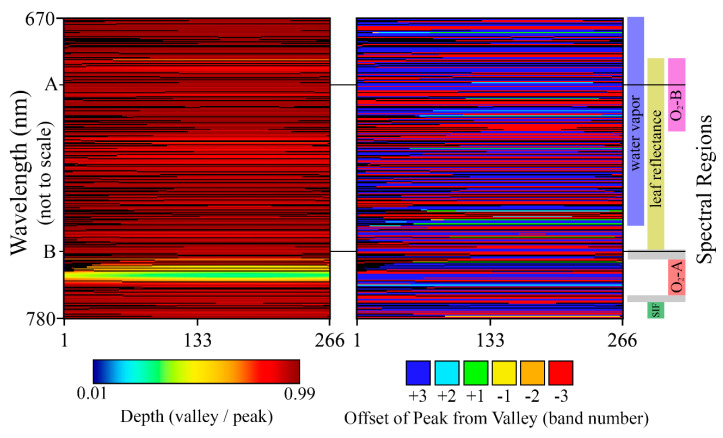
The absorption features identified in simulated FIREFLY spectra are arranged according to their wavelength (resulting in the wavelength axis being not to scale). The proportional depth (left plot) and valley to peak band distance (right plot) for each feature in each spatial pixel is shown. A black point indicates that the absorption feature was not present in that spatial pixel. The spectral regions relevant to SIF retrieval (see Figure 16) are drawn to the right of the plots, and are scaled to match the wavelengths of the absorption features.

For an indirect SIF retrieval product, several factors have been discussed that would influence the decision of whether or not to include a particular absorption feature in a particular SIF retrieval effort. Figure 20 shows that this study has provided all of the relevant information needed to pursue this potential retrieval approach with FIREFLY. Following the lines attached to the “A” and “B” annotations in Figure 20 demonstrates the multi-step decision-making process for individual absorption features. Firstly, an absorption feature has to be represented in every spatial pixel of FIREFLY to be included in a retrieval effort, as indicated by an unbroken line of color across the left plot. Secondly, the expected proportional depth of the absorption feature might be required to meet a particular standard. As part of this study, the depth of each feature has been determined separately for each FIREFLY pixel, and is displayed in the left plot. Thirdly, there could be requirements for the spectral distance between the valley and peak band for each absorption feature, again derived for each feature in each FIREFLY pixel, and displayed in the right plot. The right plot also reveals that the optimum peak band changes for some absorption features across the FIREFLY spatial pixels, which could be another factor in filtering absorption features. Fourthly, the absorption features could be included or excluded based on the spectral regions they reside in, shown to the far right of Figure 20. It can be seen that absorption feature “A” would be subject to influences from water vapor and oxygen absorption, and leaf reflectance shape, whereas feature “B” resides in a region free from additional influences.

**Figure 21:**
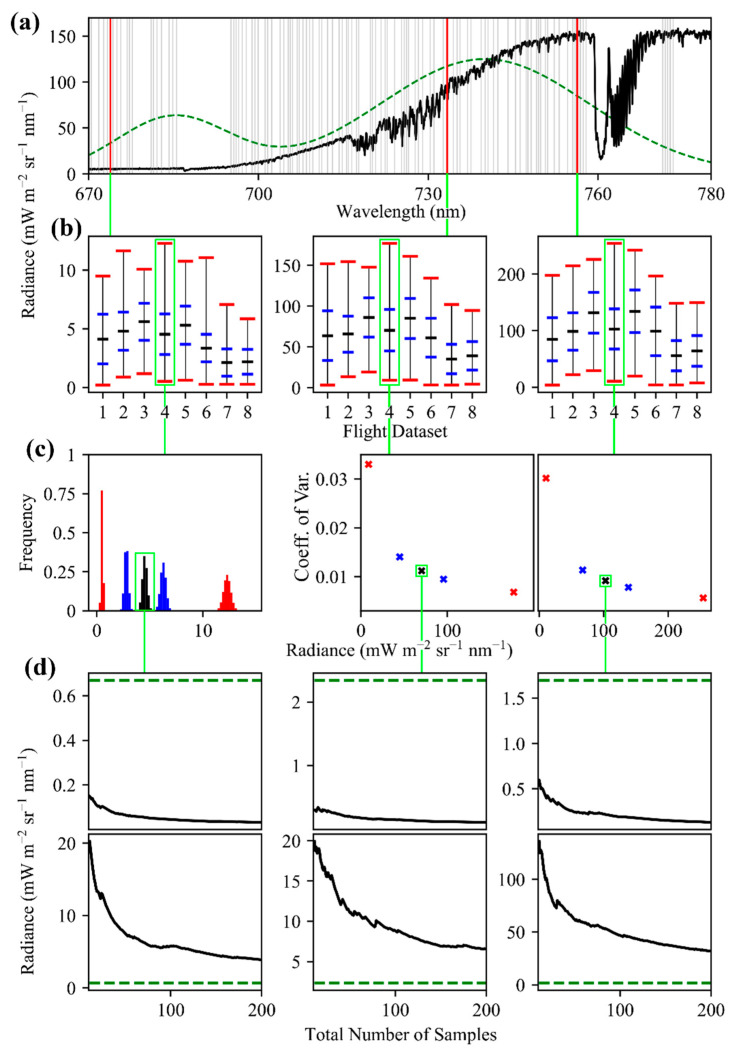
(**a**) Absorption features (gray lines) shown with a FIREFLY vegetation spectra (black line), and a simulated SIF function (green dashed line, not to scale). (**b**) Radiances from in-flight acquisitions (see Appendix A Table A2) for the valley bands of the features shown as red lines in (**a**). The radiance range (vertical black line), mean (horizontal black line), ± one standard deviation (blue lines), and minimum and maximum (red lines) are shown. (**c**) The frequency (left) and coefficient of variation (middle and right) of radiances for Monte Carlo simulation (100,000 samples) of noise distributions centered on the key values of the radiance ranges outlined in green in (**b**). Colors match those of the mean, minimum etc., in (**b**). (**d**) The 95% confidence intervals on the mean for the values outlined in green in (**c**), over the accumulation of samples from the Monte Carlo simulation. Direct retrievals from the valley bands (top row) and indirect retrievals (bottom row) are shown, along with expected SIF radiance (dashed green lines) based on the SIF function in Figure 1.

The final component of the decision-making process for forming a set of absorption features for an indirect SIF retrieval is the expected noise of the feature, relative to the expected SIF magnitude at the spectral location of the feature. To estimate the expected noise for a feature, it is necessary to have an estimate of radiance for the valley and peak bands, and to understand the relationship between noise and radiance for the instrument. The underlying principles of how noise interacts with indirect SIF retrievals have already been explored here (Figure 3 and Figure 15). To provide a frame of reference for expected target radiances, the FIREFLY in-flight acquisitions used in this study were searched for vegetated and non-vegetated areas with internally similar spectral shapes. For each of these areas, the mean, minimum, maximum and standard deviation of radiance was derived per spectral band. Across eight in-flight scenes, a total of 12,149 non-vegetated pixels and 437,617 vegetated pixels were considered, and the results were used to parameterize forward modelling of uncertainty. Figure 21 displays several examples of absorption features, showing their observed radiances in vegetated pixels from in-flight acquisitions, and demonstrating simulated noise propagation via Monte Carlo simulation.

In order to make a decision for an absorption feature based on the expected noise, there has to be a metric on which to base the decision. Various metrics and standards were proposed and tested during this study, but in Figure 21, the relationship explored is between the expected 95% confidence interval on the mean of the radiance and the expected SIF in the valley band, based on the simulated SIF function shown in Figure 1. Figure 21 demonstrates several key points. It is immediately clear that even within the limited in-flight acquisitions utilized in this study, the radiance values for individual bands varied considerably both within and between acquisitions. Since noise has a non-linear relationship with radiance (Figure 9 and Figure 21c), it is suggested that decisions about absorption features in an operational product would be made on a per-acquisition, or possibly even per-pixel basis. Additionally, Figure 21d shows that both indirect and direct retrievals can be expected to show an attenuation of noise influence with repeated independent samples, and confirms that indirect retrievals will have a much stronger response to the influence of random noise than direct retrievals (as suggested in Figure 3).

Mitigating the effects of noise by increasing sample size is a potentially promising avenue to improve indirect SIF retrievals. Generally, in spectroscopy, equivalent benefits to sample size increase are achieved by amalgamation, either spectrally (spectral binning), or spatially (spatial aggregation). Spectral binning faces some challenges for its application to indirect SIF retrievals, particularly because the expected shape of SIF is quite nuanced (Figure 1). Since variation in the properties of the SIF spectral shape are of direct ecological interest, and because they can reveal vegetation composition, condition, and stress, it would not be ideal to have to assume aspects of SIF shape in order to implement spectral binning. Additionally, the relatively narrow FWHM, and high spectral sampling resolution (0.051 nm) of FIREFLY result in closely-packed absorption features that could be obscured by spectral binning, with a mean of 2.09 absorption features per nanometer across the spectral range of FIREFLY. The high spectral sampling resolution also makes FIREFLY effective at capturing local minima and maxima to increase the proportional depth of absorption features. Simulated spectra with half the spectral sampling resolution of FIREFLY (0.12 nm) showed absorption features losing a mean of 39.83% of their proportional depth, with some absorption features losing over 90% of their depth. Even this simple look at the benefits of the spectral sampling resolution of FIREFLY advises against spectral binning as a solution to shot noise.

Spatial aggregation also encounters some interesting challenges when applied to SIF retrieval. Spatial aggregation is functionally equivalent to drawing additional random samples from combined populations with similar variances, to estimate the combined mean. However, when retrieving SIF at the spatial resolution of FIREFLY, especially in mixed land-cover and land-use areas, it is reasonable to expect that many ground targets will not contain SIF. In the context of a SIF retrieval, non-SIF-containing ground targets contain only noise. Therefore, including ground targets that do not contain SIF in a spatially aggregated SIF estimate could actually be harmful to the uncertainty of the retrieval. One approach could be to filter observations to only include those expected to contain SIF in spatial aggregations. Such filtering could be achieved for FIREFLY acquisitions by using vegetation indices such as NVDI (Figure 19). Obviously, the caveat to this approach is that it would introduce a guaranteed negative bias in the SIF retrievals, and result in a product that was actually an estimate of minimum SIF for each given area.

**Figure 22:**
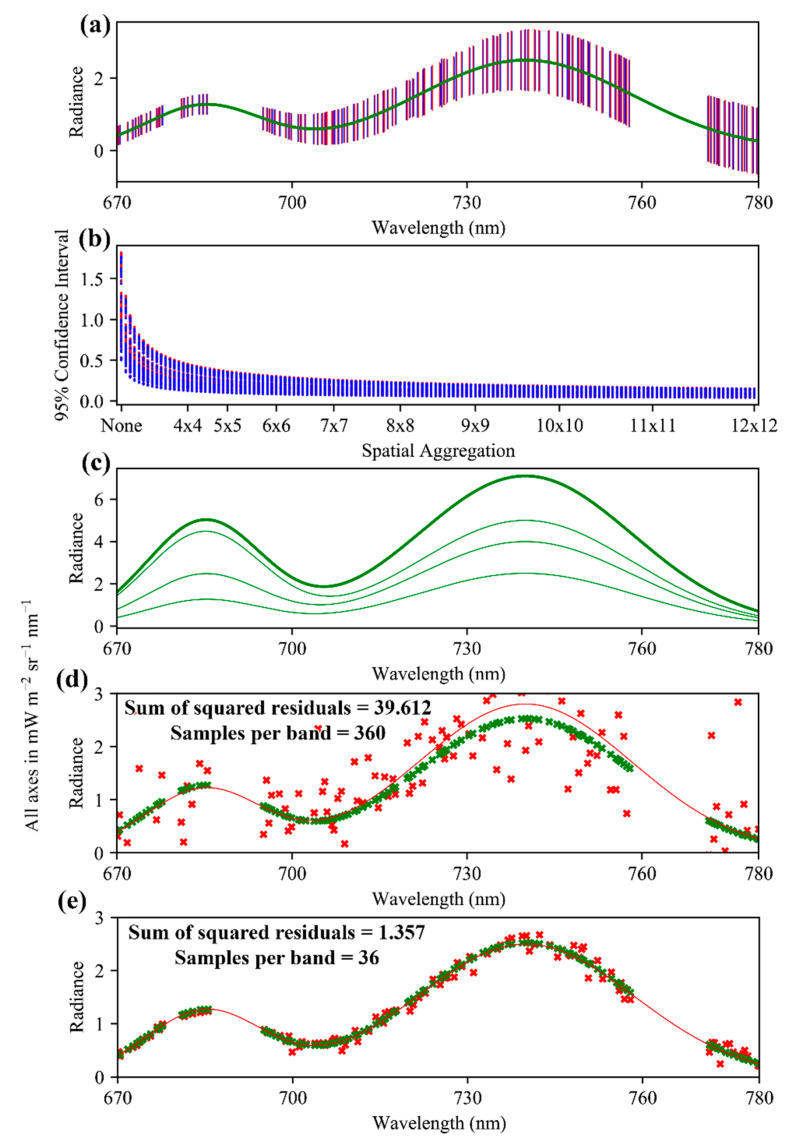
(**a**) Expected variation in valleys (red lines) and peaks (blue lines) relative to a simulated SIF signal (green line); (**b**) expected confidence intervals for valleys (red dots) and peaks (blue dots) at different spatial aggregation factors (functionally, sample sizes); (**c**) example simulated SIF functions (thin green lines) and their sum (thick green line); (**d**) indirect SIF retrievals (red x) fitted to a SIF signal (red line). The true SIF value in each band (green x) is also shown. Note that the vertical axis matches the range of (e) for the sake of comparing the fitted functions, and therefore does not show all of the residuals; and (**e**) direct SIF retrievals from valleys (red x) fitted to a SIF signal (red line). The true SIF value in each band (green x) is also shown.

Another consideration for the implementation of spatial aggregation in SIF retrievals is that the shape of the SIF signal is expected to vary even between adjacent SIF-containing pixels. In fact, some of the most desirable applications for SIF retrievals, such as monitoring of vegetation stress in agriculture or forestry, rely on the detection of such differences in SIF shape on a fine spatial-scale. Figure 22 demonstrates an indirect SIF retrieval process with FIREFLY. The selected absorption features are modelled for their expected noise (a), and combined to simulate spatial aggregation at different scales up to 12 × 12 FIREFLY pixels (b). Forward modelling of the expected noise using the information from these studies could help choose spatial aggregation factors in an operational product. In the example in Figure 22b it can be seen that the reductions in confidence interval are minimal beyond 6 × 6 aggregation. There is also separation between groups of absorption features visible in Figure 22b, as a small gap in the vertical distribution of confidence intervals at lower spatial aggregation factors, suggesting that this process could be refined to optimize the set of absorption features to those that would respond best to spatial aggregation.

Proceeding with a 6 × 6 spatial aggregation based on Figure 22b, Monte Carlo simulation was used (36 samples to represent a 6 × 6 spatial aggregation) to introduce expected noise into the absorption features. Then, for each of these simulated samples, a SIF signal with random values for the scaling factors and 740 nm peak (see Section 2.2, and see Appendix A.2 for more details) was simulated (see Figure 22c for examples) and added to the radiance values for the noise-containing valleys and peaks. To complete the process as it would occur in an operational retrieval, estimates of SIF were derived from each simulated absorption feature, and used to fit the SIF simulation function. The independent variables for the fit were the two scaling factors, and the 740 nm peak, with the sum of the squared residuals as the response variable. However, due to the amplification of uncertainty when random noise in applied to indirect retrievals, the resulting fit was extremely poor, and the spatial aggregation factor had to be iteratively increased to 60 × 60 (360 pixels) before a reasonable approximation of the true SIF function was achieved (Figure 22d, sum of squared residuals = 39.612 mW m^−2^ sr^−1^ nm^−1^). For comparison, a 6 × 6 spatial aggregation of direct SIF retrievals from just the valley bands produced an excellent fit (Figure 22e, sum of squared residuals = 1.357 mW m^−2^ sr^−1^ nm^−1^).

Although further work should be conducted to refine the understanding of noise in FIREFLY, and to formalize the required retrieval standard for particular applications, these findings suggest that indirect SIF retrievals could only reach acceptable levels of uncertainty with a large sample size. Even though this degree of spatial aggregation would reduce the granularity of FIREFLY observations substantially, in turn reducing its usefulness for certain applications such as precision agriculture, in-flight FIREFLY acquisitions could still make indirect SIF retrievals that would be comparable to most satellite retrievals of SIF, with the advantage of controlling the time and conditions of overpasses to investigate many important influences on SIF. The alternative avenue to increasing sample size for indirect retrievals would be to make repeated observations of targets over time. While this would be theoretically possible with consecutive overpasses, it would be more effective to mount FIREFLY on a static platform, such as the tower-based platforms that have shown success for SIF retrievals in other studies [42,43]. The instrument would also be suitable for other forms of mobile platform that could still facilitate high-repeat acquisitions, such as overhead trams, or unmanned autonomous vehicles [23].

It is also worth considering the potential of indirect SIF retrievals using just the oxygen absorption features. Since these features typically have extremely low radiances inside their valleys, the best ratios between SIF and noise are expected to be found there. The mean radiance of the oxygen A feature valley across vegetation targets drawn from the in-flight data in this study was 16.88 mW m^−2^ sr^−1^ nm^−1^ (±4.1 95% CI, N = 437,617), which is equivalent to an expected standard deviation of 0.4 mW m^−2^ sr^−1^ nm^−1^. For context, the amount of SIF found in the oxygen A valley, based on the function shown in Figure 1, would be 1.17 mW m^−2^ sr^−1^ nm^−1^. The proportional depth of the oxygen A feature was found to be 0.11 (±0.03 95% confidence interval), which would be quite resistant to non-uniform influences (Figure 14). However, the extreme depth of the oxygen A feature arises partly because of the relatively high radiance of the peak band, with a mean of 164.94 mW m^−2^ sr^−1^ nm^−1^ across the in-flight acquisitions, resulting in an expected standard deviation of 1.17 mW m^−2^ sr^−1^ nm^−1^. The oxygen B feature is represented in the in-flight vegetation targets with a mean proportional depth of 0.757; a mean valley radiance of 4.26 mW m^−2^ sr^−1^ nm^−1^; and mean peak radiance of 7.71 mW m^−2^ sr^−1^ nm^−1^. These low radiances in both bands give a favorable relationship between the expected standard deviations of 0.21 mW m^−2^ sr^−1^ nm^−1^ for the valley, and 0.28 mW m^−2^ sr^−1^ nm^−1^ for the peak, and the estimated SIF at the wavelength of the valley of 1.26 mW m^−2^ sr^−1^ nm^−1^.

These evaluations of indirect retrievals from oxygen features are encouraging, but there would still be considerable uncertainty when the expected noise is propagated through to a simulated SIF retrieval. Therefore, similar recommendations to those previously discussed: to mitigate noise through spectral binning, spatial aggregation, and repeated observations, can be applied to indirect SIF retrievals from the oxygen features. The one exception would be that the narrow FWHM and high resolution spectral sampling of FIREFLY provide representations of the valleys of the oxygen features with several bands each, facilitating spectral binning. The oxygen A feature is typically represented by five bands, and the oxygen B feature is represented by three bands within a 10% total variation according to assessment of the relative depth according to the in-flight vegetation targets.

Finally, indirect retrievals from oxygen features, unlike solar absorption features, require atmospheric effects to be accounted for, since the valley bands will be subject to oxygen absorption and the peak bands will not. Therefore, it is necessary to either utilize an atmospheric model parameterized with retrieval conditions, or to utilize an in-scene reference target with equivalent path-length to other targets. Each of these approaches could add uncertainty to SIF retrievals, and warrant further study. However, the high resolution (~3cm ground resolution at typical acquisition altitude) RGB camera integrated into G-LiHT could help identify vegetation-free reference targets in combination with G-LiHT’s other multispectral data [1].

#### 4.1.2. Direct Retrievals

A direct retrieval of SIF involves removing all the non-SIF components of the radiance observed in a band or bands, with the remaining energy treated as the SIF signal (see Section 2.1). Exactly which components need to be accounted for and removed, and the supplementary requirements for estimating these components, varies greatly depending on instrument properties, and by spectral region. The influences at work in various spectral regions have already been discussed in the context of indirect retrievals (Section 4.1.1 and Figure 16), but for direct retrievals the requirements are even more stringent, since every estimation of a component of a radiance measurement can contribute uncertainty to the retrieval of a SIF signal that is only a small proportion of the total radiance in any given band (Figure 1 and Figure 21).

The focus of this study was characterizing the attributes of the FIREFLY instrument itself that would influence SIF retrievals, but for the following discussions it is important to bear in mind that an operational product utilizing direct SIF retrievals would also have to account for irradiance conditions, atmospheric conditions, and target reflectivity shapes, which could contribute additional uncertainties of various types (see Figure 3). However, FIREFLY could benefit from coincident data from the other instruments mounted on G-LiHT when it comes to estimating non-SIF components for direct retrievals. For example, the downwelling radiometer could give an estimate of irradiance conditions, and the lidar could provide an accurate path-length to each spatial pixel on the ground (Figure 18).

In SIF retrieval efforts with other instruments, the challenges of using the full spectral range have been met by the use of spectral fitting methods. By utilizing explicit estimates of particular spectral components, such as atmospheric water vapor, and reflectivity profiles, or by deriving their influence implicitly through principle component analysis, or singular vector decomposition, other studies have demonstrated the ability to retrieve SIF from the amalgamated information from many absorption features [19,28,29]. Such approaches could be a promising avenue for SIF retrievals from FIREFLY, especially considering the wealth and variety of coincident data available from G-LiHT instruments, and will the subject of future studies.

In terms of FIREFLY attributes characterized in this study, it is important for direct retrievals that a thorough characterization of FWHM was performed (Figure 5), since knowledge of FWHM is essential to predict the interaction of irradiance conditions with target reflectivity profiles. There is some uncertainty remaining in the FWHM estimation which could encourage some further refinement in the characterization. The standard deviation of the residuals of the fit of the observations to the FWHM function (Figure 5) is equivalent to a relatively large potential uncertainty in SIF estimates. Although a future characterization effort with a larger number of repetitions could prove beneficial, the FWHM function in its current form seems to explain cross-track patterning observed in test SIF retrievals in in-flight acquisitions. Moreover, there is a clear heteroscedasticity in the residuals that may be inflating the apparent variation, while actually being an artefact of characterizing the spatial and spectral variation in FWHM separately.

The high spectral sampling resolution of FIREFLY (0.051 nm) is helpful for direct SIF retrievals since it can either provide a higher sample size to mitigate noise (2160 total bands), or allow very specific filtering by spectral region. The FWHM of FIREFLY, combined with the spectral sampling resolution, does lead to considerable overlap in the spectral sampling of adjacent bands. However, in spectroscopy, this is not typically treated as a problem to the assumptions of independent sampling required by some statistical analyses.

Forward modelling efforts to understand the influence of noise in potential FIREFLY SIF retrievals produced favorable results for a direct retrieval approach. Although it is a somewhat arbitrary standard, Figure 21d shows 95% confidence intervals of direct retrievals falling well within the magnitude of simulated SIF signals, even without the aid of repeated samples. Figure 22 demonstrates direct retrievals with propagated noise recovering an excellent estimation of a simulated SIF signal, albeit with some repeated sampling via simulated spatial aggregation. 

The oxygen features provide some of the best opportunities for direct retrievals. The key advantage of using oxygen features for SIF retrievals, as discussed in the previous subsection, is the expectation that there will be a comparatively high ratio of SIF to radiance, and therefore noise, in these regions. Additionally, the underlying SIF emission spectrum has peaks at 685 nm (FIREFLY band 296) and 740 nm (FIREFLY band 1372), close to the oxygen B feature at 688 nm (FIREFLY band 333), and the oxygen A feature at 761 nm (FIREFLY band 1774). Indeed, the observed radiances for the oxygen features from in-flight acquisitions, and the modelled expected noise, compared favorably to the expected levels of SIF (see Section 4.1.1).

While oxygen features are an appealing subject for an initial operational SIF product, it is also hard to detect error in direct SIF retrievals from oxygen features without experimentation in a controlled environment; robust validation data; or least a simulation environment where error can be traced back to its specific source. Obscuration of the presence and provenance of error arises because oxygen features typically contain so little total energy that retrieved estimates of SIF more frequently fall within plausible bounds for SIF values by chance alone. This tendency prompts particular caution when spectra are being processed by techniques that scale, or put bounds on radiance, such as atmospheric and reflectivity models, and is the primary reason that this study focuses on propagating error explicitly, rather than working backwards from retrieval efforts to estimates of error.

Direct retrievals with FIREFLY are further facilitated by this study with the characterization of dark current characterization, and resulting correction (Figure 8), and the characterization of response and linearity (Figure 7). Some uncertainty does remain in the fit of the response function, with the residuals having a standard deviation of 1.6 DN mW^−1^ m^−2^ sr^−1^ nm^−1^, and the following relationship with wavelength:

(7) $$U=2\times 10^{-4}\cdot W-0.1005$$

where *U* is the uncertainty in DN and *W* is the wavelength in nanometers. Over the spectral range of FIREFLY, this is equivalent to a band radiance uncertainty of between 3.5% and 5.1%, meaning that a future characterization of band response with a larger sample size could be useful.

Another essential characterization in this study was of the stray light behavior of FIREFLY. The FWHM characterization captures the influence of directly neighboring spectral regions on each band, but confirming that there were no far-field stray light effects in FIREFLY was also a particular concern. Far-field stray light would act much like a random influence (Figure 3), adding considerable uncertainty to SIF retrievals, while also being extremely difficult to detect in in-flight acquisitions. The investigation of FIREFLY with GLAMR revealed no evidence of far-field stray light (Figure 13), but it would be recommended to repeat far-field stray light checks periodically.

**Figure 23:**
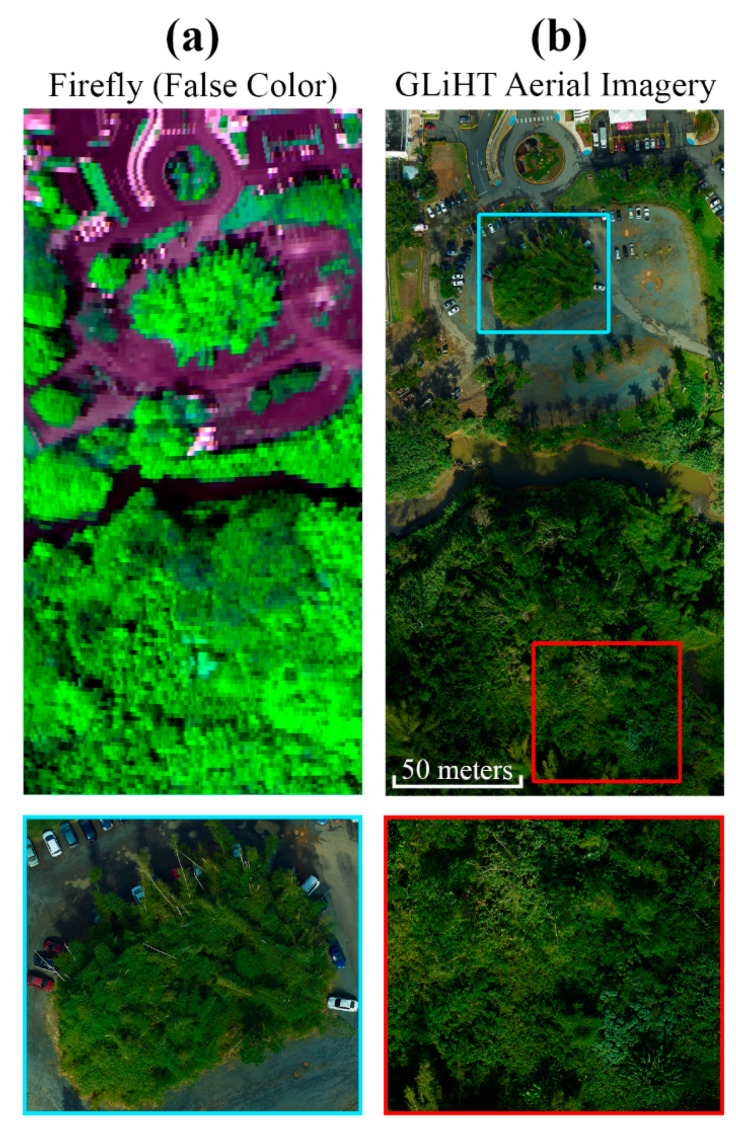
(**a**) False color RGB FIREFLY image (red: 670.2 nm, green: 780.2 nm, blue: 700.9 nm) from an in-flight acquisition in Puerto Rico, USA (greens: vegetated targets; purples/pinks: non-vegetated targets); (**b**) high resolution aerial photograph from G-LiHT RGB camera, with zoom of mixed cover (light blue box), and vegetation (red box) shown.

There are several other instrument attributes that are displayed as potential sources of uncertainty in Figure 2, but were primarily investigated initially through in-flight acquisitions in this study. One example is spatial stray light, which would arise if the point spread function (PSF) of FIREFLY extended beyond the boundaries of each spatial pixel on the detector array. An influence of this nature would again be a significant problem for SIF retrievals, as signals from neighboring ground targets would be mixed in each pixel. There is no evidence to suggest that spatial stray light is an influence in FIREFLY, with sharp boundaries between cover types visible in many FIREFLY acquisitions (Figure 18, Figure 19 and Figure 23). However, there is no substitution for a thorough characterization of the PSF, and this will be a focus of future characterization efforts.

**Figure 24:**
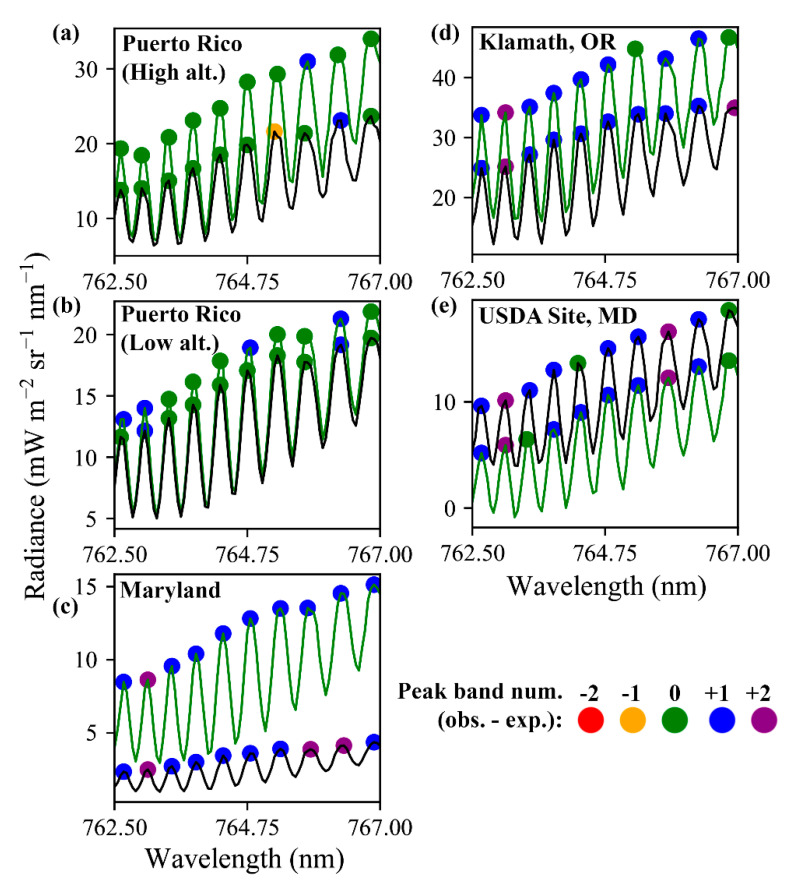
Radiances from vegetated (green line) and non-vegetated (black line) targets for absorption features found in the region of influence of the oxygen A feature. Results for five sets of acquisitions are shown (**a**–**e**), with the location annotated in each panel. The bands identified as the peak for each absorption feature are shown (circles), and colored according to their distance in bands from the FIREFLY band in which the peak most commonly occurred across all in-flight acquisitions. The Puerto Rico acquisitions were for the same area, but collected at high (**a**) and low (**b**) altitudes, respectively.

Spectral shift and squeeze describe the redistribution of the recorded wavelength of energy as a function of the temperature and pressure conditions experienced by the instrument during acquisitions. Any inconsistency in the wavelength of recorded energy would be confounding for SIF retrievals. In an effort to investigate spectral shift and squeeze, the approximate locations of peaks of ten large absorption features inside the region of influence by the oxygen A feature were established with inflection analysis (Section 2.3.1). The FIREFLY band in which the local peak for each feature occurred was then retrieved in samples from every in-flight acquisition used in this study, on the assumption that these acquisitions incorporated a range of temperature and pressure conditions. Figure 24 shows that the peak radiance for each absorption feature did move between FIREFLY bands, but that it rarely moved by more than one band from the modal peak band for each feature, suggesting random noise could be a factor. However, there were some examples found (shown in Figure 24) where the peaks moved in a consistent spectral direction, which could be evidence of a minor spectral shift. Further investigation is recommended, and will be facilitated by the tool demonstrated in Figure 24. Additionally, a more rigorous test for spectral shift and squeeze could involve mounting a panel of known reflective profile over the instrument port during in-flight acquisitions, accompanied by measurements of temperature and pressure conditions.

Spectral smile is the term used when the detected wavelength of energy shifts as a function of the spatial pixel in which it occurs, due to the inconsistent distribution of energy by the detector grating. This effect would have a serious impact on SIF retrievals, but there is no evidence that FIREFLY has a spectral smile. A spectral smile could be identified by a shift in the cumulative distribution function of energy in cross-track targets, but after correction for FWHM effects, there were no cross-track effects observable in either the controlled acquisitions of the panels (Figure 10), or the cross-track homogenous targets found in in-flight acquisitions (for example, the cross-track road in the left panels of Figure 19.

In-flight acquisitions were also used to look for a keystone effect, whereby energy intended for one set of spatial pixels would be distributed onto another, varying as a function of wavelength. Functionally, a keystone influence mixes portions of spectra from different spatial pixels, which would be extremely confounding to SIF retrievals, especially if the underlying SIF emission function is to be derived from multiple estimates across the spectral range of the instrument, or by spectral fitting methods. There is no evidence to suggest that FIREFLY has a keystone effect, but it is hard to be certain from in-flight acquisitions, as there would have to be sharp boundaries between heterogeneous targets aligned with the spatial sampling boundaries of the instrument. Although there are plenty of examples of well-defined boundaries between distinct cover types in the in-flight acquisitions (Figure 19), a check for keystone could be the subject of a future controlled acquisition, or calibration facility test.

As a final note on the characterizations of instrument attributes in this study: with the fine spectral sampling resolution of FIREFLY it was possible to observe variation in the GLAMR laser in some results. The GLAMR laser peak was observed to shift during acquisitions, and have slight inconsistencies in the tails of the spectral profile. Any samples where these effects were observed were removed from analyses, but it is an interesting perspective on the quality of the FIREFLY instrument that it served to characterize its own calibration facilities in some instances.

### 4.2. Interpretation of SIF Retrievals

Thus far, this discussion has focused on the influence of various instrument attributes and retrieval conditions on the potential quality of SIF retrievals. However, it is important to consider that once SIF retrievals are acquired with sufficient accuracy, additional information will aid in their meaningful interpretation. A SIF signal in a remote sensing observation arises because of a complex integration of many factors in the sampled area. For example, the coverage and composition of vegetation are major determining factors in the total SIF signal per pixel. The high resolution (~3 cm) RGB camera integrated into G-LiHT could certainly provide estimates of vegetation cover (Figure 23), and has already shown promise in identifying some vegetation species in work currently in progress. In addition, hyperspectral data from G-LiHT’s imaging spectroscopy could provide spectral un-mixing, if appropriate end-members for local species can be derived from coincident ground measurements or pre-existing studies.

Vegetation condition, in terms of its fluorescent light yield at the time of overpass, and the relative action of photosystems I and II, mediates the SIF signal resulting from a particular coverage and composition. The respective action of the photosystems can be expected to co-vary with phenology and diurnal cycle, as well as many potential vegetation health conditions and underlying stressors that are of ecological interest. However, to study particular influences on SIF variation, or to infer vegetation condition from SIF retrievals, repeated measurements may be required to establish and disentangle background diurnal and phenological cycles. Static platforms, overhead trams, or unmanned autonomous vehicles (UAVs) can be used to facilitate repeated observations of a target, and aid in the interpretation of airborne and satellite observations of SIF.

Vegetation structure will also influence the observed SIF in a retrieval. For example, structurally convoluted forests will show the effects of complex patterns of reabsorption and reemission. The aspects of SIF signal that can be attributed to structure can also be expected to be highly dependent on view-angle. The coincident lidar observations from G-LiHT, supported by the high-quality inertial navigation system and Global Navigation Satellite System may provide some insight into the vegetation structure and view-angle for FIREFLY SIF retrievals (Figure 18). However, it is likely that the most useful insight into the dependence of airborne SIF retrievals on vegetation structure, and its sensitivity to view angle, will arise from ongoing work to simulate SIF with ray-tracing in complex vegetation.

## 5. Conclusions

In this study, the results of an intensive characterization effort for a high performance spectrometer, FIREFLY (Fluorescence imaging of red and far-red light yield) were presented. To evaluate the instrument for its potential to retrieve solar induced fluorescence (SIF) by various methods, the quantitative characterizations included shot noise, full-width at half maximum (FWHM), response, linearity, band wavelength, stray light, and dark current. The results confirmed FIREFLY as a high-specification instrument for SIF retrieval by contemporary standards. Through forward modelling of simulated FIREFLY spectra and simulated SIF signals, it was determined that a high degree of precision and certainty in the characterizations was essential, since SIF signals are expected to comprise such a low proportion of radiance. The uncertainties remaining in the characterizations were propagated into potential SIF retrieval error, revealing several promising avenues for further study. In particular, it would be of value to have coincident ground control observations of leaf and canopy SIF for airborne data product validation purposes.

FIREFLY was found to be suitable for direct SIF retrievals in terms of noise and other instrument attributes, but to further reduce SIF uncertainty would require enhanced strategies to account for the influence of irradiance, atmosphere, and target reflectivity. Other studies have suggested the efficacy of various spectral fitting methods (SFM) for this purpose, and these methods will likely be the focus of efforts to produce an operational SIF product from FIREFLY. For indirect retrievals from absorption features, including the oxygen features, it was concluded that the effects of noise would have to be mitigated, with spatial aggregation a viable option for SIF retrievals intended for comparison to coarser resolution satellite observations of SIF. Repeated observations were identified as another alternative to mitigate noise in SIF retrievals, and could be achieved by mounting FIREFLY on alternative platforms such as towers or UAVs.

The interpretation of SIF observations can benefit from the contextualizing information provided by the coincident data from other instruments included in NASA Goddard’s lidar, hyperspectral, and thermal (G-LiHT) instrument package. The potential of the lidar, hyperspectral, and high resolution RGB imagery should be considered in the context of designing synthesized SIF data products.

Overall, the challenges of accurately retrieving SIF can only be met with a well-characterized instrument. This study represents substantial progress towards the appropriate level of characterization for FIREFLY to retrieve a competent SIF product. Several aspects of this study should be of benefit to efforts to characterize similar instruments, including the analytical tools, the forward modelling tools, and the investigative approaches employed.

## Figures and Tables

**Table 1:** FIREFLY Attributes.

Category	Attribute	Value
Spectral	Wavelength range (nm)	670 to 780
	Bands	2160
	Band interval	0.051
Spatial	G-LiHT flight altitude (m)	335
	Spatial pixels (after 6x binning)	266
	Field of view (degrees)	23.5
	Instantaneous field of view (degrees)	0.142
	Swath width (m)	140
Sensor	Type	TE-cooled sCMOS
	Dynamic range (bits)	16

**Table 2:** Goddard’s laser for absolute measurement of radiance (GLAMR) observations of FIREFLY.

Wavelength (nm)		
Step Size	Start	End
0.05	690	694.5
	707	711.5
	725	729.5
	760	764.5
0.15	690	694.5
	707	711.5
	725	729.5
	742	746.5
	742	746.5
	760	764.5
1	690	1000

## Supplementary Materials

Code and data are available at https://glihtdata.gsfc.nasa.gov/files/FIREFLY/.

## Appendix A

### Appendix A.1. Indirect Retrievals

Indirect Solar Induced Fluorescence (SIF) retrievals rely on baseline knowledge of the proportional depth of an absorption feature in the absence of SIF, derived as follows:

(A1) $$D=\frac{V}{P}$$

where *D* is the proportional depth of the absorption feature, *V* is the radiance of the valley (lower radiance) band, and *P* is the radiance of the peak (higher radiance band). This expected depth can then be used to derive an estimate of SIF from an acquisition as follows:

(A2) $$F=\frac{V_{o}-(P_{o}\cdot D)}{1-D}$$

where *F* is the estimate of SIF, *V_o_* is the observed radiance in the valley band in the acquisition, and *P_o_* is the observed radiance in the peak band. Note that this approach produces the same result if led by a prediction of the expected peak as follows:

(A3) $$F=\left(P_{o}-\frac{V_{o}}{D}\right)\cdot \left(\frac{D}{1-D}\right)$$

When modelling the various influences considered in this study, including uniform, and non-uniform sloped and random, the influence is applied to the valley and peak band separately, and the SIF estimate is made as in A2 or A3. For example,

(A4) $$F=\frac{\left(V_{o}+I_{v}\right)-\left(P_{o}+I_{p}\right)\cdot D}{1-D}$$

where *I_v_* is the value of the influence at the wavelength of the valley band, and *I_p_* is the value of the influence at the wavelength of the peak band.

### Appendix A.2. SIF Signal Simulation

When a simulated SIF signal was required it was expressed as the sum of three normal distributions of radiance, such that the relative SIF across all the bands of FIREFLY can be expressed as

(A5) $$SIF_{rel}=\overset{780}{\underset{w=670}{\sum }}\left(PSI_{w}\cdot SF_{PSI}\right)+\left(PSIIa_{w}\cdot SF_{PSII}\right)+PSIIb_{w}$$

where *SIF_rel_* is the value of SIF in relative units; *PSI* is a normal distribution representing the action of photosystem I, centered at 740 nm, with a standard deviation of 20 nm; *PSIIa* and *PSIIb* are normal distributions representing the action of photosystem II, centered at 685 nm and 740 nm, respectively, and with standard deviations of 10 nm and 18 nm, respectively; *SF_PSI_* is a scaling factor for photosystem I; and *SF_PSII_* is a scaling factor for PSII. The total radiance value for SIF can then be derived as

(A6) $$SIF_{rad}=\frac{A}{\left(\underset{670\leq w\geq 780}{\max}SIF_{rel}\right)}\cdot SIF_{rel}$$

where *SIF_rad_* is the value of SIF in radiance units; and *A* is the value of SIF in radiance units for the apex of the 740 nm peak. SIF derived from a particular wavelength would be

(A7) $$SIF_{w}=\left(\left(PSI_{w}\cdot SF_{PSI}\right)+\left(PSIIa_{w}\cdot SF_{PSII}\right)+PSIIb_{w}\right)\cdot \frac{A}{\left(\underset{670\leq w\geq 780}{\max}SIF_{rel}\right)}$$

where *SIF_w_* is the SIF estimate in radiance units for wavelength *w.*

### Appendix A.3. Modelling Tools and Code

The forward modelling tools used in this project were developed in Python 3.6, and the input data processing and relationship between the modules and scripts to produce the figures are detailed below. Please note that due to a known issue with the “matplotlib” module, the figure scripts cannot run in the latest version of Python at the time of writing (3.8.3). The forward modelling and figure production for this study was conducted in a virtual environment running Python 3.6.

The modules, scripts, and input files are available at (https://glihtdata.gsfc.nasa.gov/files/FIREFLY/). Many of the tools could be adapted to evaluate the specifications of an instrument other than FIREFLY. The Instrument_Preparation.py script contains steps to build an instance of the instrument class (initialized from Instrument_Builder.py). Note that the script will create a python file in the local directory that will act as a module to call functions for instrument specifications. The steps for creating an instrument are annotated in detail in the script. For additional information, including assistance in adapting the tools for a different instrument, please contact the authors.

In general, input data were processed in the appropriate bespoke software into a level 1 input for the Python model. Where additional standardization was needed (field headers, for example), a level 0 output from the software was created as an intermediary, then adapted into a level 1 input file. A python script for each source of input data (*_Input_Preparation.py in the model files) ingested the level 1 inputs and converted them into amalgamated JavaScript Object Notation (JSON) files. These JSON files then acted as the baseline inputs called for the subsequent analyses and visualization. In other words, all of the modules, analyses, and figure scripts can be run using the provided JSON files. The JSON files can also be imported using the functions found in the Data_Loader.py module, if the save path is updated for the user’s local environment. The prerequisite modules to install are “json”, “numpy”, “matplotlib”, “math”, “shutil”, “importlib”, and “sys”.

Below is an alphabetical list of the tools and modules available in the supplied code package, including a brief description for each:

- Absorption_Feature_Peaks.py contains a function “get_abs_feat” to return a set of absorption features and their relative depths. Additionally, contains a function “get_abs_feats_set” to return a set of absorption features filtered by spectral region, spatial pixel, or maximum band offset between valleys and peaks.
- Data_Loader.py contains a function “load_instrument” that creates an instance of the instrument class, and then uses the method to import a saved JSON file for an instrument into the instance. Additionally, contains a function “load_data” that loads specific JSON data files based on which other function calls it. There is a function “load_*” for each major dataset that imports the JSON file for the dataset via the “load_data” function.
- ENVI_File_Import.py contains a function “envi_read” that imports an ASCII file exported from ENVI as a Python dictionary.
- Instrument_Builder.py contains the class “Instrument” to represent an instrument for SIF retrieval in analyses. The class contains methods “import_instrument” to import the instrument from a saved JSON; “export_instrument” to export the instrument to a JSON, which is called on initialization of an instance of the class; “normcdf” to return a proportion from a normal cumulative distribution function for a given location of a normal distribution based on a given mean and standard deviation; “convolve” to sample high resolution reference spectra according to instrument specifications; “simulate_spectra” to simulate a set of spectra covering all the spatial pixels of the instrument; “abs_feat” to derive a set of absorption features from simulated spectra; “get_band_wl” to return the wavelength of a band in nanometers based on an input band number; and “get_band_num” to return a band number based on an input band wavelength.
- Keys_As_Sorted_Floats.py contains the function “get_key_floats” that returns a list of band wavelengths as floats and a list of bands as strings based on an input set of dictionary keys. Additionally, contains the function “get_key_ints” that returns a list of band wavelengths as integers and a list of bands as strings based on an input set of dictionary keys.
- SIF_Estimate.py contains the function “integrate_SIF” to return a range of wavelengths for an evaluation of SIF, an estimate of the absolute SIF in each of a list of input bands, and an estimate of the total SIF across all of the input bands. Additionally, contains the function “get_sos_residual” that takes an estimated SIF signal and an expected SIF signal and returns the sum of the squared residuals between the two. Additionally, contains the function “estimate_sif” that returns the values for SIF at as set of wavelengths, and the residual based on the best fit of an input set of SIF estimates to the SIF simulation key parameters (see Appendix A.2) as independent variables, and the sum of the squared residuals as the response variable.
- SIF_Simulation.py contains the function “simulate_SIF” to provide a SIF signal derived according to Appendix A.2. Additionally, contains the function “get_sif” to provide a SIF estimate at a given wavelength based on a simulated SIF signal.
- Spectral_Regions.py contains a function for each spectral region “in_*” to test whether a given band is in the spectral region in question, returning a True or False. Additionally, contains a function “all_regions” that returns a dictionary with keys named for each spectral region, and values of True or False to indicate whether a given band is in the spectral region.
- Wavelength_Band_Conversion.py contains the functions “get_bands_from_wl” and “get_bands_from_nums” that both return lists of band numbers and band wavelengths as floats, based on input lists of wavelengths and band numbers, respectively.

Below is an alphabetical list of the scripts that conducted processing and analysis, including a brief description for each. The list excludes those scripts that prepared inputs (*_Input_Preparation.py) and those that produced figures (Figure_*.py):

- Absorption_Feature_Search.py executes a series of methods on an instance of the instrument class to establish a set of absorption features for each spatial pixel found in the simulated spectra. The saved JSON file for the instrument is then updated.
- Shift_Squeeze_Test.py prints a count of flags based on analysis of an input ASCII file exported from ASCII. The flags are based on analysis of the absorption features in the trailing region of the oxygen A feature, and return a yellow flag when the apex of a feature is found ± 1 band from the expected band, and a red flag when the apex is found ± 2 bands from the expected band.
- Simulate_Spectra.py executes a series of methods on an instance the instrument class to simulate spectra based on each spatial pixel. The saved JSON file for the instrument is then updated.

**Table A1:** Filenames for figures generated from Python scripts.

Figure Number	Python Script
1	Figure_A.py
3	Figure_I.py
5	Figure_B.py
6	Figure_N.py
7	Figure_L.py
9	Figure_K.py
10	Figure_E.py
11	Figure_D.py
12	Figure_C.py
13	Figure_M.py
14	Figure_R.py
15	Figure_G.py
16	Figure_H.py
17	Figure_J.py
20	Figure_O.py
21	Figure_P.py
22	Figure_Q.py
24	Figure_F.py

**Table A2:** In-flight acquisitions used in this study.

Collect Name	ID	Location	Filename	Notes
Klamath OR	1	Oregon, USA	Klamath_OR	Outside Klamath Falls
Puerto Rico (High alt.)	2	Puerto Rico, USA	PR_BG_high_2k	High flight altitude
Puerto Rico (Low alt.)	3		PR_BG_low_2k	Low flight altitude
El Verde, Puerto Rico,	4		PR_EV2_9k	El Yunque National Forest
El Verde, Puerto Rico 2	5		PR_EV2_11k	El Yunque National Forest
Maryland	6	Maryland, USA	UAS_2017	University of Maryland UAS Test Site
USDA Site, MD	7		USDA_OPE_0	USDA Test Site
USDA Site, MD 2	8		USDA_OPE_1k	USDA Test Site
